# “Tomorrow Never Dies”: Recent Advances in Diagnosis, Treatment, and Prevention Modalities against Coronavirus (COVID-19) amid Controversies

**DOI:** 10.3390/diseases8030030

**Published:** 2020-08-06

**Authors:** Partha Laskar, Murali M. Yallapu, Subhash C. Chauhan

**Affiliations:** 1 Strathclyde Institute of Pharmacy and Biomedical Sciences, University of Strathclyde, 161 Cathedral Street, Glasgow G4 0RE, UK; laskarpartha@gmail.com; 2 Department of Immunology and Microbiology, School of Medicine, University of Texas Rio Grande Valley, McAllen, TX 78504, USA; 3 South Texas Center of Excellence in Cancer Research, School of Medicine, University of Texas Rio Grande Valley, McAllen, TX 78504, USA

**Keywords:** coronavirus, COVID-19, controversies, biology, diagnosis, treatment, prevention, technology, data, alternatives

## Abstract

The outbreak of novel coronavirus disease (2019-nCoV or COVID-19) is responsible for severe health emergency throughout the world. The attack of severe acute respiratory syndrome coronavirus 2 (SARS-CoV-2) is found to be responsible for COVID-19. The World Health Organization has declared the ongoing global public health emergency as a pandemic. The whole world fights against this invincible enemy in various capacities to restore economy, lifestyle, and safe life. Enormous amount of scientific research work(s), administrative strategies, and economic measurements are in place to create a successful step against COVID-19. Furthermore, differences in opinion, facts, and implementation methods laid additional layers of complexities in this battle against survival. Thus, a timely overview of the recent, important, and overall inclusive developments against this pandemic is a pressing need for better understanding and dealing with COVID-19. In this review, we have systematically summarized the epidemiological studies, clinical features, biological properties, diagnostic methods, treatment modalities, and preventive measurements related to COVID-19.

## 1. Introduction

**Figure 1:**
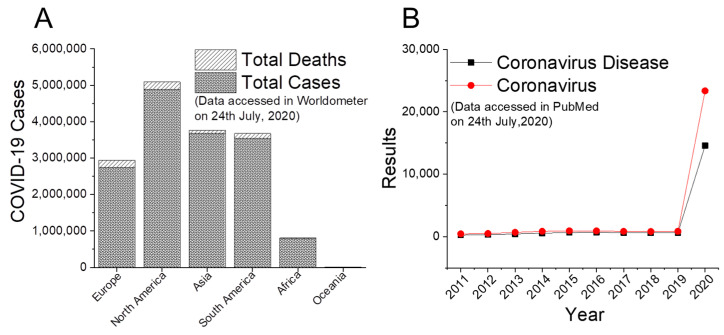
(**A**) Distribution of COVID-19 cases over various continents according to Worldometer (data accessed on 24th July, 2020). (**B**) Year-wise results for research on “Coronavirus” and “Coronavirus Disease” keyword for last 10 years (2020–2011) according to PubMed (data accessed on 24th July, 2020).

The emergence and spread of 2019 novel coronavirus (2019-nCoV) or the severe acute respiratory syndrome (SARS) coronavirus 2 (SARS-CoV-2) has threatened global public health. The coronavirus disease 2019 (COVID-2019), which is a transmission of SARS-CoV-2 to humans, was reported first in Wuhan, Hubei province, China in December 2019. Later, COVID-19 rapidly spread worldwide creating a pandemic as there have been around 15,656,884 reported cases and 636,576 reported deaths to date (24 July 2020) affecting 213 countries and territories around the world and two international conveyances (https://www.worldometers.info/coronavirus/). North America, South America, and Asia are the most affected (maximum number of cases) continents by this pandemic so far (https://www.worldometers.info/coronavirus/, data accessed on 24 July 2020) (Figure 1A). In spite of rapid development of knowledge, precautionary measurements, and clinical trials, researchers, regulatory bodies, and government administrations are facing a great challenge globally in various aspect to prevent COVID-19 pandemic. The present situation is leading us to a still unknown future and conflicting paradox in the battle against SARS-CoV-2. Further, a plethora of documents have been published on “coronavirus” and “coronavirus disease” (year-wise keyword search for last 10 years in PubMed on 24 July 2020) research, where a maximum and a very large number of results were obtained in 2020 on both the keywords in comparison to previous years (Figure 1B). Nature Index has also reported a hugely increasing global research publishing phenomenon on COVID-19 pandemic as evidenced by 66,883 articles and 19,420 preprints (https://www.natureindex.com/news-blog/the-top-coronavirus-research-articles-by-metrics). A thorough and cumulative scientific knowledge about the progress against COVID-19 amid various conflicts and controversies is the need of the time to set a clear directive in this battle. Through this review article, we provide an overview of updated and rapidly evolving progress against COVID-19 pandemic including various conflicts to the readers in a comprehensive manner. Considering this, we have summarized diverse research areas covering the current known biological properties of SARS-CoV-2, diagnostic tools for detection, therapeutic measurements for possible treatment, and prevention techniques to stop further spreading of this pandemic.

## 2. Origin, Transmission, and Symptoms

### 2.1. Origin

#### 2.1.1. Genesis, Structure, and Features

**Figure 2:**
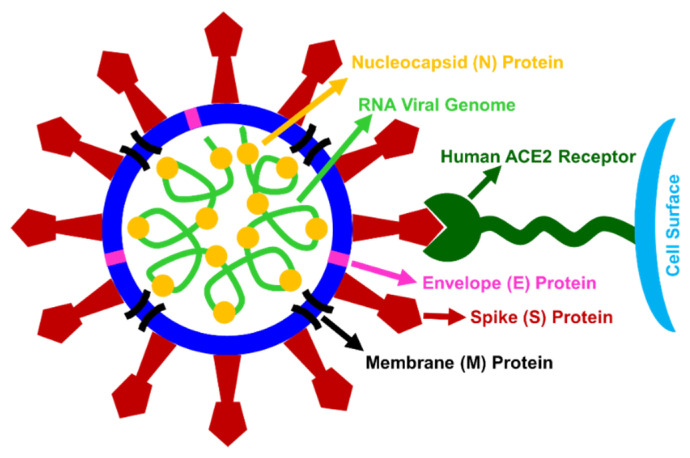
Hypothetical cartoon presentation of morphology, structural constituents, and possible receptor mediated cellular binding of SARS-CoV-2. Size, shape, and integral components are for visualization purpose only and do not exactly replicate the ultra-structural morphology of virus.

Coronaviruses (subfamily: Coronavirinae, family: Coronaviridae, order: Nidovirales) are enveloped single stranded positive sense RNA genomes that range in size from 26 to 32 kilobases [1,2]. Coronavirus consists of four structural proteins: the nucleocapsid, envelope, membrane, and spike forming a core-shell morphology (Figure 2), whose diameter is in the range from 60 nm to 140 nm with spike like projections on its surface [2,3,4]. The name, coronavirus (Latin: Corona = Crown) came out due to the presence of a crown or the sun’s corona-like spike (S) glycoprotein on viral surface forming club-shaped protrusions, which is also evidenced through the electron microscope [2,3,4]. This transmembrane spike (S) glycoprotein on viral surface mediates the entry into host cells, forming homotrimers protruding from the viral surface [5]. The receptor binding domain (RBD) in the spike (S) glycoprotein is the most mutable part of the coronavirus genome leading to generation of new properties and ability of virus to infect new cell types or even new species [6]. Based on phylogenetic relationships and genomic structures, the subfamily Coronavirinae is divided into four genera (α-CoV, β-CoV, γ-CoV, and δ-CoV). α-CoV and β-CoV only infect mammals, whereas γ-CoV and δ-CoV infect generally birds and sometimes even infect mammals. β-CoV and γ-CoV are responsible for respiratory diseases in humans and gastroenteritis in animals [2,7]. Presence of four corona viruses (HKU1, NL63, 229E and OC43) have been found in human circulation which are generally cause mild respiratory disease [8].

#### 2.1.2. SARS-CoV-2

The 2019-nCoV is phylogenetically closely related to bat SARS-like coronaviruses, hence name SARS-CoV-2 and belongs to β-CoV genus lineage B [9]. Regarding pathogenicity and transmissibility, SARS-CoV-2 may differ from other known SARS-CoV due to a significant change in its spike glycoproteins (ORF8, and ORF3b) [10]. Doremalen et al. [11] showed that the SARS-CoV-2 virus remained viable in aerosols (<5 μm) for at least up to 3 h and was more stable on plastic and stainless steel than on copper and cardboard. SARS-CoV-2 was first identified from the samples (cultured human airway epithelial cells along with the virus from isolated bronchoalveolar lavage fluid) of adult COVID-19 patients in Wuhan, China. Its morphology was analyzed by negatively stained sample under transmission electron microscopy (TEM) [12]. Zhu et al. [12] made a conclusion based on TEM ultra-structural image of SARS-CoV-2 virus particles. This follows as (i) it is generally spherically shaped with a diameter ranges from 60 to 140 nm, (ii) it has an envelope with quite distinctive 9 to 12 nm protein spikes, and (iii) it has genetic material which matched to the genome from lineage B of the genus β-CoV—showing more than 85% identity with a bat SARS-like CoV (bat-SL-CoVZC45, MG772933.1). These observations are similar to the overall structure of Coronaviridae family viruses. TEM image of an isolate from the first United States case of COVID-19 is also evidenced the spherically shaped viral particles (colorized blue) containing cross-sections through the viral genome (black dots) [13]. The crystal structures of the unliganded SARS-CoV-2 main protease (M^pro^) and its complex with an α-ketoamide inhibitor was used to provide some knowledge about the drug target for COVID-19 [14]. This M^pro^ enzyme is essential one along with the papain-like protease(s) for processing the viral RNA translated polyproteins. Thus, inhibition of this enzyme may block the viral replication. The three-dimensional crystal structure, at 1.75 Å resolution, of the SARS-CoV-2 M^pro^ is highly similar to the SARS-CoV M^pro^, due to the 96% sequence identity. Dimerization (necessary for catalytic activity) of the M^pro^ is regulated by Domain III (residues 198 to 303) mainly through a salt-bridge interaction between Glu^290^ of one protomer and Arg^4^ of the other.

### 2.2. History

Like SARS-CoV-2, world also has seen an outbreak of worldwide pandemic and a large-scale fatal swine disease during the past two decades because of three other zoonotic (transmitted from animals to human) coronaviruses, such as SARS in 2003, Middle East Respiratory Syndrome (MERS) in 2012, and Swine Acute Diarrhea Syndrome (SADS) in 2017. Surprisingly, these viruses have been originated from bats, a natural source of various other highly lethal zoonotic viruses (such as Hendra, Nipah, Ebola, and Marburg viruses). SARS and SADS viruses were claimed to be originated in China [15,16]. Based on these facts, scientists including Chinese research group from Wuhan (primary epicenter of COVID-19) also warned further possible coronavirus outbreaks from bats and with a high probability that outbreak will occur in China [17].

### 2.3. Transmission

#### 2.3.1. Unknown Intermediary Host

Both SARS-CoV and MERS-CoV bat β-coronaviruses crossed over to humans through an intermediary host, which was palm civet cats in the Guangdong province of China (SARS-CoV, in 2002–2003, mortality rate 11%) and dromedary camels in Saudi Arabia (MERS-CoV, in 2012, fatality rate 34%), respectively [18]. In the case of COVID-19, the virus was transmitted to human from bats, but the intermediary host animal(s) are not yet known. A study claimed that the intermediary host animal is pangolin due to the following findings on Pangolin-CoV, SARS-CoV-2, and BatCoV RaTG13 viruses: (i) At the whole-genome level, both Pangolin-CoV and SARS-CoV-2 share 91.02% similarity among them, (ii) Pangolin-CoV and SARS-CoV-2 are reported to be the second closest relative to each other than to BatCoV RaTG13, (iii) In the receptor binding domain (RBD) of spike glycoprotein, five key amino acid residues involved in the interaction with human angiotensin converting enzyme 2 (ACE2) of Pangolin-CoV and SARS-CoV-2 are consistent, and (iv) Only SARS-CoV-2 contains a potential cleavage site for furin proteases unlike both Pangolin-CoV and RaTG13 [19]. It was also concluded that the transmission of human SARS-CoV-2 virus from bat may include more than one intermediary host including pangolins [20].

#### 2.3.2. Human to Human Transmission

Similar to SARS-CoV, the 2019-nCoV is reported to have the capability to transmit efficiently among humans due to familial cluster of pneumonia [9]. Several cases were reported person-to-person transmission of this virus not only through family settings, but were also in hospital and infected travelers [9,21]. Person-to-person transmission of the SARS-CoV-2 infection is occurred via airborne droplets to the nasal mucosa in closed environments, close contact between people, unwashed hands, and touching contaminated surfaces with less possibilities. Within the incubation period ranges from 2 to 14 days in general, SARS-CoV-2 may replicate locally in cells of the ciliated epithelium resulting cell damage and inflammation. Primarily, respiratory secretions of any infected person are used to diagnose the presence of virus by special molecular tests including normal/low white cell counts with elevated C-reactive protein (CRP) [18]. Additionally, abnormal computerized tomographic (CT) chest scan is also proved to be helpful to diagnose any infected person even for those with no symptoms or mild disease [18]. The SARS-Cov-2 showed lower mortality but faster spreading than SARS-CoV and MERS-CoV. Isolation of SARS-CoV-2 from oral swabs, bronchoalveolar lavage fluid, and stool proved them to be highly contagious [22,23].

#### 2.3.3. Receptor-Mediated Cellular Entry

SARS-CoV-2 infects human by interacting with a functional receptor, metallopeptidase named angiotensin converting enzyme 2 (ACE2), for its successful cellular entry (Figure 2) [22,24]. Crystal structure of the C-terminal domain of spike protein in complex with human ACE2 (hACE2) revealed an overall similar binding mode as that of SARS-CoV with hACE2 [24]. It was determined that 2019-nCoV uses ACE2 as a cellular entry receptor in human, Chinese horseshoe bats, civets, and pigs but not for mice and cells without ACE2 protein expression capability [22]. Other coronavirus receptors, such as aminopeptidase N and dipeptidyl peptidase 4 do not play any role for cellular entry of 2019-nCoV [22]. Previous studies revealed that almost all human organs are known to have ACE2 mRNA, though the protein expression of ACE2 mRNA was largely unknown. Such ACE2 receptor is found to be present in arterial and venous endothelial cells, arterial smooth muscle cells in the lungs, stomach, small intestine, liver bile ducts, colon, skin, kidney parietal epithelial cells, lymph nodes, and in the brain [25]. The surface of lung alveolar epithelial cells and enterocytes of the small intestine also express ACE2 protein allowing them to be infected by SARS-CoV-2 [25]. The tissues of the upper respiratory tract are not the primary site of entrance for SARS-CoV, as oral and nasal mucosa and nasopharynx did not show ACE2 expression on the surface of epithelial cells, rather upper respiratory tract might be susceptible to secondary infections from the infected lower respiratory tract. Lower lungs may show higher opacity in the CT scans due to its more ACE2 expression [26]. Higher viral loads have been recorded in the nose than the in throat, with similar viral loads seen in asymptomatic and symptomatic patients [27].

### 2.4. Symptoms and Impact

Coronaviruses generally are found to cause acute and chronic respiratory, enteric, and central nervous system diseases in humans as well as in other animals. The symptoms of a COVID-19 patient are usually fever, cough, sore throat, breathlessness, fatigue, and feeling of discomfort. For most of the people, it is found mild. For elderly and the patient with comorbidities may develop pneumonia, acute respiratory distress syndrome (ARDS), and multi-organ dysfunction leading to death. Many infected people are found to be asymptomatic causing a problem for early detection and controlling the spread of disease. Mortality rate estimated by the World Health Organization (WHO) (as of 3 March 2020) is 3.4% (https://www.worldometers.info/coronavirus/coronavirus-death-rate/). Further, speculation about the association of human coronaviruses with more serious human diseases (such as multiple sclerosis, hepatitis, or enteric disease in infants) are still under question due to no proper evidence [8].

## 3. Diagnostic Modalities

In absence of any approved therapeutics or vaccines for the treatment of COVID-19, WHO has promoted “test, isolate, and trace” method as a preventive measure. Thus, early, rapid, and accurate diagnosis of COVID-19 patients is becoming very crucial to control the sources of infection and to prevent further community spread. With a gradual understanding of biological properties of SARS-CoV-2, various diagnostic methods and device strategy with point of care facilities have been developed for COVID-19 detection worldwide. A summary of various diagnostic methods (Table 1) are presented for the COVID-19 detection. Various countries approved and implied different testing methods according to the regulation of their own health agencies based on situation and availabilities. Below subsections are summarized the recent developments on diagnostic methods based on (i) nucleic acid, (ii) protein, (iii) chest scan and (iv) autopsy.

### 3.1. Nucleic Acid-Based Detection

Nucleic acid-based detection strategy has been widely used against detection of various diseases, including coronavirus and recent COVID-19. In this section, we review some nucleic acid-based detection methods that are commonly being employed for the diagnosis of COVID-19.

#### 3.1.1. Polymerase Chain Reaction

Polymerase chain reaction (PCR) is an enzymatic method widely used in molecular biology to make millions to billions of copies of a specific DNA sample [28]. This method involves following steps in a series or cycles of temperature changes: (i) Denaturation: separating the two strands of the DNA containing the gene segment with the application of heat, (ii) Annealing-marking gene segment of each strand of DNA with a primer, (iii) Primer extension: using a DNA polymerase to assemble a copy alongside each segment, and (iv) Repeat: continuously copy the copies [28]. Various PCR-based methods are an indispensable, common, and rapid techniques for scientist to amplify a minute nucleic acid sample to a large enough amount for a number of applications [29]. Due to high sensitivity and high sequence specificity, the PCR-based method has been used as a routine and reliable technique for detecting coronaviruses. Coronavirus is a RNA virus, so in general reverse transcriptase-PCR (RT-PCR) method is implied as follows: coronavirus RNA is transcribed into cDNA by reverse transcription, then the PCR is performed on cDNA, and finally detection of PCR product through specific detection method(s) (gel visualization and sequencing) [30,31].

**Real-time:** Real-time reverse transcriptase-PCR (RT-PCR) detection method is evolved as a common platform for detection of all kinds of coronaviruses due to its low cost per test, less time-consuming process and more sensitive than the conventional RT-PCR assay [32,33]. The whole genome sequence of SARS-CoV-2 enabled to develop PCR-based kits to diagnose COVID-19 in laboratory and clinical settings [34,35,36,37,38,39]. Corman et al. [34] developed a robust diagnostic methodology considering the SARS-related virus sequences available in GenBank. A close genetic relatedness to the 2003 SARS-CoV and synthetic nucleic acid technology helped this process to design and validate such strategy without using any virus isolates and samples from infected person. Such a technique can successfully discriminate 2019-nCoV from SARS-CoV. This approach provided the first version of the diagnostic protocol to the WHO from exclusivity testing on 75 clinical samples (13 January 2020). A real-time RT-PCR based test is found to be more sensitive than radiological test for pediatric patients [36]. Pediatric patients with milder symptoms, showed no clear clinical signs or chest X-ray findings but their real-time RT-PCR exhibited positive results. Further, in this report, real-time RT–PCR showed positive results in rectal swab-testing but negative results in nasopharyngeal swab-testing for eight out of ten pediatric patients suggesting shedding of virus in the gastrointestinal tract and a possible fecal-oral transmission. Wang’s group [37] reported that the RT-PCR based findings using different types of clinical specimens collected from 82 infected individuals. In their study, it was found that viral loads were significantly correlated among 30 pairs of throat swab and sputum samples. Overall, real-time RT-PCR based method enables developing a high-throughput testing for rapid, on-demand, low-cost, reliable, quantitative detection technique against COVID-19 in clinical settings [39].

**Probe free:** A team of Indian Institute of Technology, Delhi, India reported first probe-free real time PCR assay for COVID-19 detection (http://www.iitd.ac.in/content/icmr-approves-probe-free-covid-19-detection-assay-developed-iit-delhi-0). They have used comparative sequence analyses to identify unique regions (short stretches of RNA sequences) in the SARS COV-2 genome, which are not present in other human coronaviruses. In this highly sensitive assay, primers can specifically target unique regions (conserved in over 400 fully sequenced) of COVID-19 genomes, which was reported after extensive optimization using synthetic DNA constructs followed by in vitro generated RNA fragments. Indian Council of Medical Research has approved this technique as it does not require any fluorescent probes (thus low-price) but still useful for high throughput testing.

#### 3.1.2. Isothermal Nucleic Acid Amplification

Isothermal amplification of nucleic acids is a rapid, efficient, and alternative amplification technique than PCR. This process can be applied at a constant temperature without any thermos-cycling apparatus, unlike in the case of PCR [40,41]. The isothermal amplification technique can be performed in water bath, on the cell surface, or even inside living cells, making it a superior technique over PCR [40,41]. Based on reaction kinetics of isothermal nucleic acid amplification, it is divided to exponential amplification, linear amplification, and cascade amplification. These are further sub-divided into transcription mediated amplification, nucleic acid sequence-based amplification, signal mediated amplification of RNA technology, strand displacement amplification, rolling circle amplification, loop-mediated isothermal amplification of DNA, isothermal multiple displacement amplification, helicase-dependent amplification, single primer isothermal amplification, and circular helicase-dependent amplification, based on the developments in molecular biology of DNA/RNA synthesis [40,41]. Furthermore, the use of microfluidic chips, capillary platforms, and test paper with isothermal amplification technique has been developed for single-cell or single-molecule analysis. Among these, loop-mediated isothermal amplification (LAMP) has been implied successfully for coronavirus detection [42,43,44,45]. LAMP technique can amplify target nucleic acid sequence using two or three sets of primers and a polymerase at a constant temperature (~60–65 °C) [46,47,48]. In comparison to PCR-based technique, LAMP can produce considerably higher amount of DNA with high strand displacement and replication activity due to the use of additional pair of “loop primers”.

Park et al. [46] developed reverse transcription LAMP (RT-LAMP) assay(s) to detect genomic RNA of SARS-CoV-2. These RT-LAMP assays (in combination with leuco crystal violet colorimetric detection method) can detect as low as 100 copies of SARS-CoV-2 RNA within 30 min. These RT-LAMP assays were highly specific towards SARS-CoV-2 compared to other human coronaviruses (hCoV-229E, hCoVOC43, MERS-CoV, and SARS-CoV). Yu et al. [49] also developed a rapid and sensitive isothermal LAMP based method (iLACO) for the detection of COVID-19 virus RNA or cDNA samples. In this method, iLACO was used to amplify a fragment of the ORF1ab gene using 6 primers, which was proved to be specific for SARS-COV-2 species (i.e., low chance for false positives) in comparison to the sequences of 11 related viruses by the help of online tool Primer-BLAST (including 7 similar coronaviruses, 2 influenza viruses and 2 normal coronaviruses). iLACO can detect synthesized RNA equivalent to 10 copies of 2019-nCoV (performance is comparable to Taqman based qPCR detection method), where reaction time varied from 15–40 min based on virus load in the collected samples. Another LAMP-based colorimetric detection method was reported to identify SARS-CoV-2 virus RNA from purified RNA or cell lysis (without an RNA purification step) [48]. The sensitivity of this portable method is equivalent to a commercial RT-qPCR test with only heating and visual inspection. Zhu et al. [47] demonstrated a successful and accurate diagnosis of COVID-19 using one-step RT-LAMP coupled with nanoparticles-based biosensor (NBS) assay (RT-LAMP-NBS) within approximately 1 h (from sample collection to result interpretation). They have employed two designed LAMP primer sets (F1ab-RT-LAMP and np-RT-LAMP), heating block (to maintain a constant temperature at 63 °C), a real-time turbidity (LA-320C) and visual detection reagents (VDR) in addition to NBS interpretation to simultaneously amplify and detect genes of SARS-CoV-2 in a “one-step” and “single-tube” reaction. The sensitivity of SARS-CoV-2 RT-LAMP-NBS was 12 copies (each of detection target) per reaction, whereas no cross-reactivity was observed for all pathogens of non-SARS-CoV-2 (virus, bacteria, and fungi). The RT-LAMP-NBS assay showed 100% the analytical sensitivity of SARS-CoV-2 for oropharynx swab samples of clinically diagnosed COVID-19 patients and 100% specificity for clinical samples collected from non-COVID-19 patients.

#### 3.1.3. CRISPR Diagnostics

CRISPR-Cas (clustered regularly interspaced short palindromic repeats-CRISPR associated) is an adaptive immune system, which was discovered first in *Escherichia coli* in 1987 and later also in other bacteria species. These are found predominantly in archaea (87% of genomes) than in bacteria (50% of genomes) [50,51]. Being an immune system of archaea and bacteria, CRISPR and CRISPR-associated proteins deliver protection against invasive nucleic acids (such as DNA, or RNA from phages, plasmids, and other exogenous DNA elements) [50,51]. Scientists later exploited this immune responsive system by reengineering to target parts of genetic material for precise genetic alterations of any particular cellular type, which is the basis of CRISPR therapeutic and diagnostic platforms for human [52,53]. This adaptive immune system is also widely used as a tool for SARS-CoV-2 detection. CRISPR associated enzyme Cas13 has already been utilized for rapid and portable sensing for successful RNA-targeting [54]. A Specific High-sensitivity Enzymatic Reporter unLOCKing (SHERLOCK) platform was developed by combining isothermal preamplification with Cas13 to detect single molecules of RNA or DNA for Dengue or Zika virus [55]. An updated SHERLOCK protocol has been reported for multiplexable, portable, rapid, and quantitative COVID-19 detection (https://broad.io/sherlockprotocol), which can target sequences in a range between 20 and 200 aM (10-100 copies per microliter of input). Another development of accurate CRISPR-Cas12-based lateral flow assay able to detect SARS-CoV-2 with 95% positive predictive agreement and 100% negative predictive agreement from respiratory swab RNA extracts (less than 40 min) [56]. Another newly developed method, SARS-CoV-2 DNA Endonuclease-Targeted CRISPR Trans Reporter (DETECTR), was found to perform simultaneous reverse transcription and isothermal amplification by (i) RT-LAMP for RNA extracted (for nasopharyngeal or oropharyngeal swabs), (ii) Cas12 detection of predefined coronavirus sequences, and (iii) cleavage of a reporter molecule confirms, which detects the virus [56]. A FnCas9 Editor Linked Uniform Detection Assay (FELUDA) was developed for detecting nucleotide sequences, classifying nucleobase identity, and inferring zygosity [57]. FELUDA is able to distinguish clear signatures of SARS-CoV-2 sequence in synthetic DNA within one hour using a specific ribonucleoprotein (RNP) from non-specific RNP (such as H1N1 or HBB). FELUDA can also clearly distinguish between two SARS-CoV-2 and SARS-CoV-1 sequences. This approach further can be developed as lateral flow assay on a paper strip to distinguish SARS-CoV-2 synthetic DNA using SARS-CoV-2 specific RNP.

### 3.2. Protein-Based Detection

Protein-based testing has become as an alternative and additive detection strategy in addition to nucleic-acid based testing methods for coronavirus [58]. In response to any infected viral protein antigens, antibodies (i.e., a blood protein produced in response to and counteracting a specific antigen) are generated in patient’s body resulting a very specific antigen-antibody (Ag-Ab) serological interaction. Detection of this specific antibody level(s) due to the SARS-CoV-2 infection can be useful for surveillance of COVID-19 pandemic. This indirect serological test opens up wide range of possibilities, such as, (i) successful detection of asymptomatic patients, (ii) creating large a window of testing time even with a gradual decrease of viral load, (iii) protect community transmission due to false negative results by other methods, and (iv) proper guidance for individual quarantine period [59,60,61,62,63,64,65,66]. Kwok-Yung Yuen’s group [61] successfully detected antibodies generated in response to SARS-CoV-2 viral proteins at the time when the detection of the viral proteins become difficult due to gradual declining trend of viral load(s). Serological test using enzyme-linked immunosorbent assay (ELISA) for antibodies (immunoglobulin M, IgM and immunoglobulin G, IgG) is more confirmatory and unreliable results from oral swabs for 2019-nCoV detection [62]. This test can be applied for respiratory, blood, or fecal samples. Guo et al. [63] conducted a COVID-19 profiling study on early humoral response based on IgA, IgM, and IgG response. This study found that IgM and IgA antibody were detected 5 days, while IgG was detected 14 days after symptom onset, with a positive rate of 85.4%, 92.7%, and 77.9%, respectively [63]. The detection of IgM by ELISA was found more efficient than that of qPCR after 5.5 days of symptom onset [63]. A successful immunological field-effect transistor (FET)-based biosensing device was developed for detecting SARS-CoV-2 in clinical samples, where the sensor was developed by conjugating a specific antibody against SARS-CoV-2 spike protein to graphene sheet coated FET [64]. This rapid diagnostic device for SARS-CoV-2 antigen requires no sample pre-treatment or labelling. This is a highly-sensitive detection method for the SARS-CoV-2 spike protein at concentrations of 1 fg/mL in phosphate-buffered saline and 100 fg/mL in clinical transport medium, 1.6 × 10^1^ pfu/mL in culture medium and 2.42 × 10^2^ copies/mL in clinical samples. The false positive results in serological tests for COVID-19 is a concern due to the presence antibodies generated against other coronaviruses (such as for common cold) irrespective of the presence of SARS-CoV-2 antibodies. Recently, researchers have got a high frequency of cross-reactivity in plasma samples from 15 COVID-19 patients against the S protein of SARS-CoV-2 and SARS-CoV [65]. However, more accurate antibody-based detection method can be made with additional features of infected patient in serological test, such as (i) combined (IgM and IgG) antibody assay rather than a single antibody test, (ii) more number of testing, (iii) report of elevated levels of C-reactive protein, D-dimer, lymphocytes, leukocytes, or blood platelets [66].

### 3.3. Chest Scan-Auxiliary Test

There are growing concerns regarding the COVID-19 testing despite of huge efforts in this direction. Due to sudden outbreak and a huge increase of COVID-19 cases, a sufficient number of COVID-19 test kits are unavailable in hospitals and healthcare centers. Further, an automatic detection system with a quick diagnostic capability could be an alternative or auxiliary method to prevent community spreading of COVID-19. In this situation, most countries have recommended the RT-PCR-based methods as the standard technique for COVID-19 diagnosis. Serological tests are also considered to be the primary technique for COVID-19 detection. However, small hospitals, health centers in sub-urban and village areas, even private hospitals in sub-urban area may not have an approved RT-PCR testing center or PCR testing infrastructure facilities. On the other hand, chest-scan, a routine technique implied for prominent pneumonia pattern, has been evolved as useful non-invasive technique for COVID-19 detection [67]. Both chest X-ray and computed tomography (CT)-scan were successful to distinguish the manifestations of typical pneumonia in the case of MERS-CoV and SARS-CoV infection [68,69]. These scans have helped to diagnoses suspected person to isolate and treat more quickly, even when the RT-PCR based test did not respond properly. Such tests have proven lung histology (lung damage or holes/honeycomb-like appearance) of COVID-19 patients [70]. Thus, chest-scan is useful for suspected COVID-19 patients with negative RT-PCR result.

#### 3.3.1. X-ray

X-rays, a form of high-energy electromagnetic radiation, are shorter wavelengths than UV rays and longer wavelengths than gamma rays. X-ray machines are widely available sophisticated diagnostic imaging technique for body, bone and other dense objects that can block the radiation through a limited exposure to radiation. In addition, X-ray scans can be used for lung infections, pneumonia and tumors. An automatic prediction of COVID-19 was successfully reported using Chest X-ray images and a deep convolution neural network based pre-trained transfer models (ResNet50, InceptionV3 and Inception-ResNetV) [71]. These pre-trained transfer models helped to obtain a higher prediction accuracy for small X-ray dataset. This model has end-to-end structure without manual feature extraction and selection methods, where the ResNet50 is an effective one among all pre-trained models in the small dataset (50 COVID-19 vs. 50 Normal).

#### 3.3.2. Computed Tomography

Computed tomography (CT) scan is a computer-assisted medical imaging device which combines cross-sectional (tomographic) scanned images of specific areas or virtual slices of any organ taken from different angles producing a 3D view of that particular organ. CT-scan is one of the methods used to diagnose various abnormalities of the chest (such as pneumonia, lung cancer etc.) [72,73]. Thus, chest CT-scan is also being used as a fast, painless, non-invasive and accurate auxiliary diagnostic method in addition to the RT-PCR test for the suspected COVID-19 patient [74,75,76]. The National Health Commission of China included the chest CT findings as evidence of clinical diagnosis of COVID-19 for patients in Hubei province at the fifth edition of the Diagnosis and Treatment Program of 2019 New Coronavirus Pneumonia due to the false-negative rate of RT-PCR test for COVID-19 patient [74]. Several groups found chest CT scan more sensitive and better diagnostic tool in comparison to RT-PCR for COVID-19 detection [75,76]. Recent investigations demonstrated that the CT-scan of COVID-19 patient(s) clearly showed bilateral pulmonary parenchymal ground-glass and consolidative pulmonary opacities, with a rounded morphology, crazy-paving pattern, linear opacities, and peripheral lung distribution [77,78]. In contrast, other study did not find any lung cavitation, discrete pulmonary nodules, pleural effusions, and lymphadenopathy [77]. Further, high-resolution CT (HRCT)-scan for the chest is reported to be important tool to help clinicians to diagnose quickly and accurately the effected lung disease [79]. Artificial Intelligence (AI) and deep learning-based automated CT image analysis of lung have been also developed to distinguish COVID-19 pneumonia and Influenza-A viral pneumonia [80,81,82]. These automated deep learning based methods can produce graphical pattern of a particular COVID-19 patient which is helpful for clinician to diagnose prior to pathogenic testing [83]. In spite of such clinical diagnostic values, CT scan still fails to come at the forefront of COVID-19 diagnosis due to the following reasons: (i) it is expensive, (ii) it requires technical expertise, and (iii) it is incapable of distinguishing SARS-CoV-2 pneumonia from other viral pneumonia and hysteresis.

### 3.4. Autopsy-On Demand

An autopsy report, through examination of a corpse by dissection, is an important source of information for research purposes to evaluate any disease causing death. Autopsy has been proved to be hugely beneficial to diagnose emerging and reemerging infectious diseases, like COVID-19 [84,85,86]. In contrary, several groups raised their concern that the few autopsies have been performed on patients who died with suspected or confirmed COVID-19 infection especially in the primary epicenters of pandemic (such as China and Italy) [85,87]. Despite the suggestion by WHO on performing post-mortem examinations for COVID-19 deaths with following recommended safety procedures, many Governments including Italy discouraged the practice of autopsy during the period of increasing number of death and even some scientific report highlighted that the post-mortem examination does not have any primary diagnostic role, whereas autopsy may still have a clinical role in selected cases [88,89]. Though based on autopsies, physicians can determine a profound change of the view of COVID-19 disease not as a pneumonia but a systemic, vascular disease, putatively generated by autoimmunity [85]. Thus, a strong recommendation was urged to perform full autopsies on patients who died with suspected or confirmed COVID-19 infection with recommended exceptional biosafety guidelines to reduce the further spread of potential infection from any corpse.

## 4. Treatment Modalities

**Figure 3:**
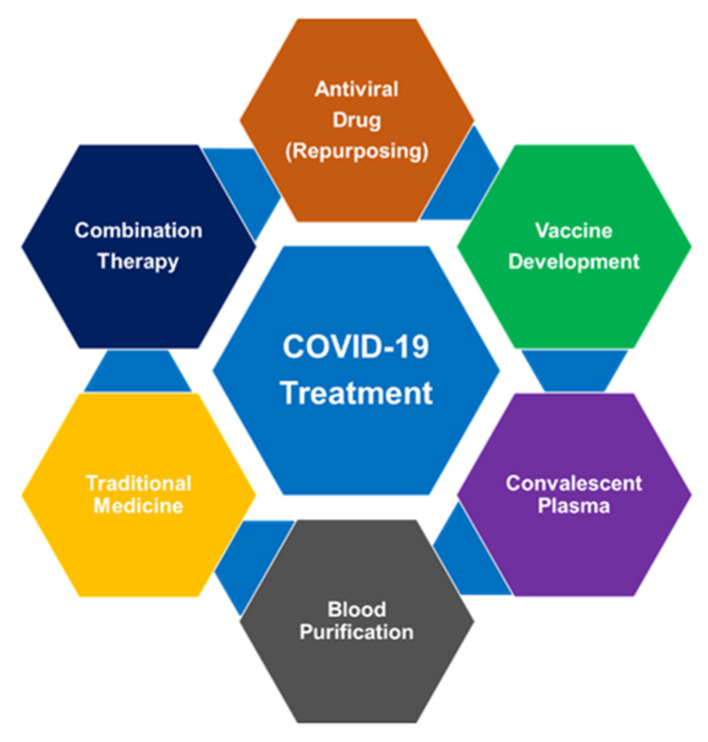
Possible treatment modalities against COVID-19.

There is currently no clinically proven therapeutic regimen to prevent and eradicate SARS-CoV-2 infection [90]. COVID-19 is being managed by the supportive treatment (oxygenation and ventilation, conservation fluid management). However, the use of broad-spectrum antibiotics [91]. This section summarizes various treatment modalities for COVID-19 (Figure 3).

### 4.1. Antiviral Drugs

Viral infection is always a major concern for morbidity and mortality in animals and humans worldwide. Development of antiviral drugs have been always a pressing need to treat such viral infections. Since the approval of first antiviral drug, idoxuridine in 1963, 90 drugs were clinically approved to treat nine human infectious diseases (human immunodeficiency virus, HIV; hepatitis B virus, HBV; hepatitis C virus, HCV; herpesvirus; influenza virus; human cytomegalovirus; varicella-zoster virus; respiratory syncytial virus; and human papillomavirus) [92]. The antiviral drugs mostly inhibit the viral development rather than destroying the target pathogen unlike most antibiotics. A broad-spectrum antiviral is found to be effective against a wide range of viruses based on drug repurposing strategy [93].

**Figure 4:**
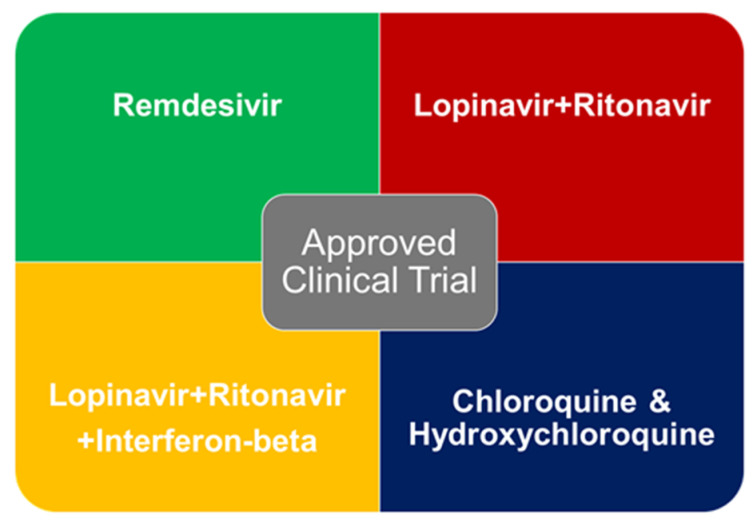
Four most promising drug candidates or combinations approved for clinical trial for COVID-19 treatment by WHO (announced on 18 March 2020).

Drug repurposing or drug repositioning is a cost-effective and time-efficient alternative strategy, which involves the recycle or re-use of clinically approved drugs for new disease instead of searching of new drugs [94,95]. In contrary to in vitro phenotypic screening of known drugs, in silico/computational drug repurposing strategy is a hypothesis-driven approach to identify the drugs for the treatment of any disease using big data analysis [96,97]. The drug-repurposing has been implied for several human diseases including antiviral drug development against coronavirus [95]. Various groups have proposed number of drug candidates through drug-repurposing (in vitro and in silico) for COVID-19 treatment [98,99,100,101]. WHO focused and initiated the “SOLIDARITY Trial” (announced on 18 March 2020) of four existing antiviral compounds/formulations (Figure 4) to assess their clinical benefit against COVID-19 [102,103].

Remdesivir, an antiviral compound, which showed activity against multiple variants of Ebola virus in cell-based assays and rhesus monkey model. Chloroquine (CQ) and its derivative hydroxychloroquine (HCQ), antiviral compound(s) have been used to treat malaria and amebiasis. A combination of lopinavir and ritonavir, is co-formulated for HIV-1 treatment. Another combination of lopinavir and ritonavir plus interferon-beta (LPV/RTV-IFNb) has been approved for the treatment of relapsing–remitting multiple sclerosis and secondary progressive multiple sclerosis. About 115 clinical trials were identified by Belhadi et.al., which includes open-label studies (46%), double-blind (13%), and single blind studies (10%) [104]. They also classified the number of trials (*n*) and total numbers of planned inclusions (*N*) for lopinavir/ritonavir (*n* = 15, *N* = 2606), chloroquine (*n* = 11, *N* = 1102), hydroxychloroquine (*n* = 7, *N* = 1048), and remdesivir (*n* = 5, *N* = 2155).

In contrary, several controversial reports including toxic side effects on these promising candidates under clinical trial(s) raised some challenging questions for researchers. Those have been presented below:

1. A report presents that patients (with symptom duration of 10 days or less) receiving remdesivir showed clinical improvement than those receiving placebo, but it did not make any statistically significant clinical benefits [105].
2. An open-label non-randomized clinical trial study demonstrated significant decrease in viral load and carriage duration in COVID-19 patients receiving hydroxychloroquine (600 mg/day during ten days). This treatment showed enhanced effects in combination with azithromycin, but it identified serious methodological flaws [106,107]. Another randomized clinical study did not make any difference in recovery rates upon hydroxychloroquine treatment in 30 COVID patients [108]. However, a hype on CQ and HCQ has created drug shortages and affected other potential treatments (such as for patients with Lupus).
3. There was no significant benefit (clinical improvement) observed with lopinavir–ritonavir treatment [108]. Mortality and percentages of patients with detectable viral RNA at various time points were similar in the lopinavir–ritonavir group and the standard-care group. It was also reported median time to clinical improvement was shorter by one day for lopinavir–ritonavir group than that observed with standard care.

### 4.2. Vaccine Development

Vaccination is one of the most effective and preventive medications against various diseases caused by pathogens (such as virus or bacteria). Currently there are about 25 approved vaccinations available against various life-threatening diseases, including measles, polio, tetanus, diphtheria, meningitis, influenza, typhoid, and cervical cancer (https://www.who.int/topics/vaccines/en/). A vaccine typically contains an agent (weakened or killed forms of any microbe, its toxins, or one of its surface proteins), which though resembles a disease-causing microorganism, but provides active acquired immunity to that particular infectious disease [109,110]. An antiviral vaccine helps to boost our natural immune response to an invading virus by priming it to recognize viral antigens. In general, antiviral vaccines can be classified as follows: (i) inactive or live-attenuated viruses, (ii) virus-like particle (VLP), (iii) viral vectors, (iv) protein-based, (v) DNA-based, and (vi) mRNA-based vaccines [110,111]. Like many other diseases, the vaccine development is not successful and conclusive for coronavirus disease. Until now, there is no proper vaccine is developed or approved for the treatment of human coronavirus diseases (such as SARS-CoV and MERS-CoV) [111,112,113]. Most big pharmaceutical companies also in the race to develop effective vaccines for CoV infection [112,113]. The existing knowledge on previous strategies for CoV vaccine developments can benefit the ongoing research as sequence analysis of the SARS-CoV-2 genome showed close relation to SARS (80%) and to one bat RaTG13 SARS-like CoV (96%) than to MERS CoV (54%) [112]. Liu et al. [111] reported that 188 patents (mentioned in CAS content collection) are directly associated with anti-SARS and anti-MERS vaccines (15 patents on inactive and live-attenuated virus vaccines, 28 patents on DNA vaccines, 21 patents on viral vector vaccines, 13 patents on VLP vaccines, and three patents on mRNA vaccines) with a demonstrated immune response, which could be a huge boost for COVID-19 vaccine developers. An accelerated evaluation of next-generation vaccine for COVID-19 has been triggered as soon as the genetic sequence of SARS-CoV-2 is published on 11 January 2020 [112,113,114,115]. As of 28 July 2020, 164 vaccine candidates have been identified (https://www.who.int/who-documents-detail/draft-landscape-of-covid-19-candidate-vaccines), of which 25 candidate vaccines are in clinical evaluation (Table 2) and 139 candidate vaccines are in preclinical stages.

Associated problems and pitfalls are surely a major concern resulting huge roadblock for vaccine discovery against COVID-19. The pitfalls are many folded, which as follows: (i) antibody-dependent enhancement (ADE) of viral replication for vaccinated people in future recurrence due to immune backfiring, (ii) rouge immunization resulting a huge damage on someone’s own immune cells (such as neutrophil and basophil), (iii) in addition to immune response malfunctioning, three imperatives of vaccine effort: speed, manufacture and deployment at scale, and (iv) global access for a newly developed vaccine [116,117].

Further, new findings and hypotheses stirred up the debate on COVID-19 research making it difficult as well as relevant not only in therapy but also in producing a life-saving vaccine, without any risk of generating immunity-based complications.

(i) Molecular mimicry: two independent groups (Lucchese and Flöel; Macario and Cappello) proposed molecular mimicry as the culprit in determining multi-organ damages (including anosmia, leukopenia, and vascular damage) in COVID-19 patients [118,119,120]. Indeed, there are confirmations that a number of human proteins share common epitopes with SARS-CoV-2 proteins and these epitopes are highly immunogenic. (ii) Biphasic infection: due to (a) the ability of human coronaviruses to cause respiratory re-infections, regardless of pre-existing humoral immunity and (b) evidence on circulation of severe acute respiratory syndrome coronavirus type 2 (SARS-CoV-2) in Italy before the detection of first COVID-19 case, an hypothesis was given on biphasic infection, the immunological result of a prior viral infections either by SARS-CoV-2 or different strains of coronaviruses, or potentially even other respiratory viruses resulting increased susceptibility to more severe forms of COVID-19, following a secondary infection with SARS-CoV-2 [121]. This theory is sustained by anecdotal clinical reports of “biphasic infection” and “cytokine storm” through a possible ADE immunological mechanism, which was already observed with infections sustained by other coronaviruses (such as SARS-CoV and MERS-CoV) or other viruses (such as West Nile Virus and Dengue).

### 4.3. Convalescent Plasma (Antibody) Therapy

Convalescent plasma (CP) therapy involves the administration of antibodies to a susceptible person to prevent or treat an infectious disease providing an immediate immunity [122]. In this therapy, donated blood from the infected person who’ve recovered from that infection have antibodies in their blood that can fight against the infection. The CP therapies have been tested since 1890s as a possible alternative and/or auxiliary treatment including SARS-CoV, Ebola, Influenza A (H5N1) etc. viruses [123,124]. Additionally, CP therapy did not show any serious and immediate adverse effects in earlier CoV treatment [125]. The plasma with antibodies is prepared after separation of blood cells from the donated blood which can be extended to combat against COVID-19. CP therapy has been generally approved as experimental treatment for patients with critical conditions.

Duan et al. [126] reported potentially improved clinical outcomes for 10 severe COVID-19 patients with a dose of 200 mL of CP (derived from recently recovered donors with the neutralizing antibody titers above 1:640) with additional use of antiviral agents and maximal supportive care. This study confirmed a number of positive outcomes, including, (i) neutralizing antibody level increased rapidly up to 1:640 in five cases, while for other four cases maintained at a high level (1:640), (ii) increased oxyhemoglobin saturation within three days, (iii) increase in lymphocyte counts (0.65 × 10^9^/L vs. 0.76 × 10^9^/L), (iv) decreased C-reactive protein (55.98 mg/L vs. 18.13 mg/L), (v) varying degrees of absorption of lung lesions within seven days, (vi) undetectable viral load in seven patients who had previous viremia. Additionally, treatment with human immunoglobulin is reported to increase same-day thrombotic event risk significantly (0.04 to 14.9%) [127]. Transfer of blood substances may include inadvertent infection with another infectious disease agent and react to serum constituents resulting immunological reactions such as serum sickness [128]. More high-quality studies, adequate selection of donors with high neutralizing antibody titers, central blood banking facilities to process the donated serum, efficient serological and virological assays, production and the use of CP according to precise ethical and controlled conditions are needed for implementing this therapy further.

### 4.4. Auxiliary Blood Purification Treatment

Latest studies showed that ACE2, the key functional receptor of SARS-CoV-2, is highly expressed in kidney (nearly 100 times higher than in lung) resulting it to be a main target organ for SARS-CoV-2 attack [129]. Thus, novel coronavirus infections hugely damage the kidney of any severe COVID-19 patient suffering from an immunological damage due to a cytokine storm, the imbalance of pro-inflammatory and anti-inflammatory factors. Thus, auxiliary continuous blood purification could be an essential technology to take care of inflammatory factors, cytokine storm, electrolyte imbalance, and acid-base balance for any COVID-19 patient resulting a reduced renal work-load and a possible recovery of renal function [130,131]. At present, extracorporeal blood purification technology is supplied for severely ill patient with novel coronavirus pneumonia as an auxiliary treatment to improve condition [132].

### 4.5. Traditional, Complementary, and Alternative Medicine

There is an increasing awareness, tendency, and agenda to use traditional (T) medicine (indigenous health traditions) and complementary and alternative medicine (CAM) (methods outside the biomedical mainstream, particularly in industrialized countries). Globally, half the population uses T/CAM as a preventive measure [133,134]. The National Center for Complementary and Integrative Health (NCCIH) of the National Institute of Health (NIH), USA, has included various medical methods under the umbrella term CAM, such as homeopathy, naturopathy, Ayurveda, medicinal systems, and products originating from traditional medicine [135]. Further, herbalism, aromatherapy, acupuncture, massage, and reflexology are also various name and forms among the most popular branches of CAM [134]. The potential use of CAM (sometimes along with conventional medicine) has been reported to be efficient therapeutic strategy against several virus associated diseases such as influenza, dengue, Japanese encephalitis, hepatitis C, zika, and HIV [134,135,136]. Considering the efficacy of CAM against coronaviruses with minimal reported adverse effects on host cells, Ministry of Ayush (Ayurveda, Yoga & Naturopathy, Unani, Siddha and Homoeopathy), Government of India, recommended scientists, researchers and clinicians to pursue research and use of Indian herbal drugs on COVID-19. Through a memorandum, Government of India is now trying to utilize the Ayurveda, Siddha, Homeopathy, and Unani system of medicine including prophylactic measures, intervention during quarantine, asymptomatic and symptomatic cases, public health research, survey, laboratory-based research etc. (https://www.ayush.gov.in/). Indian traditional medicinal systems, one of the oldest treatments in human history (existed nearly 5000 years ago witnessed and scripted in ancient literature), played a significant role in encountering global health due to its antiviral, anti-inflammatory and antioxidant properties [137,138]. Such a traditional branch of science could be a potential option against COVID-19 because of its proved efficacy not only in treatment but also in preventive strategy for several diseases, including respiratory viral infections through immunity boosting, rejuvenating lifestyle, and dietary management [137,138]. AYUSH recommended selected traditional drugs for COVID-19 as follows: http://ayush.mp.gov.in/sites/default/files/CORONA%20ADVISORY_3.pdf.

Furthermore, Vimalanathan et al. reported strong activity of several Indian medicinal plants in Tamil Nadu against mouse corona virus (a surrogate for human SARS-CoV), which are as follows: “Gymnema sylvestre R. Br. (Asclepiadaceae), Pergularia daemia (Forsskal) Chiov. (Asclepiadaceae), Sphaeranthus indicus L. (Asteraceae), Cassia alata L. (Caesalpiniaceae), Evolvulus alsinoides L. (Convolvulaceae), Clitoria ternatea L. (Fabaceae), Indigofera tinctoria L. (Euphorbiaceae), Abutilon indicum G. Don. (Malvaceae), Vitex trifolia L. (Verbenaceae), Clerodendrum inerme (L.) Gaertn (Verbenaceae), and Leucas aspera Spr. (Lamiaceae)” [139]. Traditional Chinese medicine (TCM) is also recently being included as one of the treatment modalities in China for COVID-19. Previous record on efficacious TCM against respiratory tract infectious diseases, such as Lianhua Qingwen capsules (exerts independent antiviral effects) and ShuFeng JieDu capsules (synergistic antiviral effects with Western medicine) on influenza viruses, has encouragd the practice of TCM against COVID-19 [140,141]. Wang et al. [142] reported massive improvement for four COVID-19 patients as a result of combination therapy including Chinese and Western antiviral medicine, where they used lopinavir/ritonavir (Kaletra®), arbidol, and Shufeng Jiedu Capsule (SFJDC) with necessary supportive care. However, lack of high-quality research, standard clinical trials, sufficient research articles, clear mechanism of action, rigorous population studies, prospective business model are also urgently required to establish the therapeutic effect and implementation of T/CAM among mass population. In spite of some emphatic results through traditional, complementary, and integrative products, practices, and practitioners against COVID-19, North American and European governments are keeping their silence on these practices (https://www.cdc.gov/coronavirus/2019-ncov/prevent-getting-sick/prevention.html) and rather warned of possible harm and overselling (https://www.fda.gov/news-events/press-announcements/coronavirus-update-fda-and-ftc-warn-seven-companies-selling-fraudulent-products-claim-treat-or). John Weeks, Editor-in-Chief of The Journal of Alternative and Complementary Medicine, mentioned this double standard attitude as he pointed out that “no practices have definitive evidence for benefit against COVID-19, yet providers with other stripes are using experimental practices and off-label drugs every day in their desperate to ease patient suffering and elicit hope” [143].

## 5. Preventive Modalities

**Figure 5:**
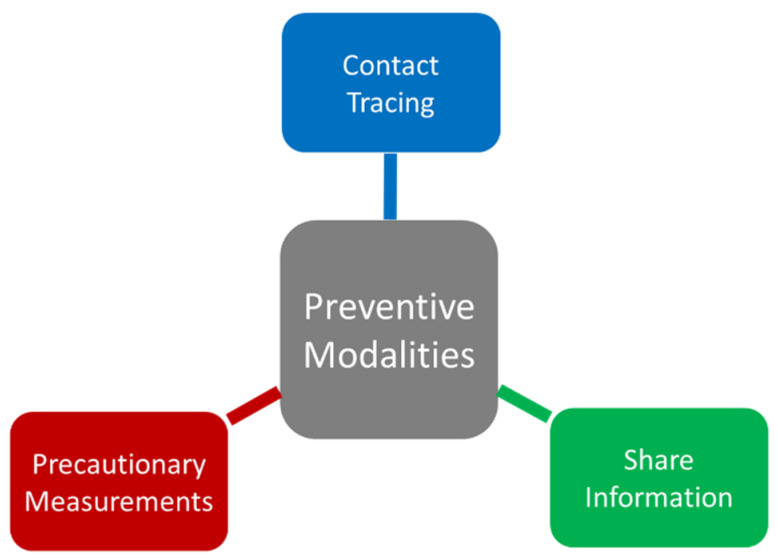
Three primary modes of preventive strategies to manage COVID-19 pandemic.

Prevention is the utmost important strategy to fight against COVID-19 in the present situation. Several preventive measurements are taken to reduce the spread and transmission of the COVID-19 infection. This is classified as follows (i) contact tracing, (ii) sharing or proper dissemination of information, (iii) precautionary measurements (Figure 5).

### 5.1. Contact Tracing and Share of Information

Track, trace, and share of the information on COVID-19 are major preventive steps to stop spreading of SARS-CoV-2. A thorough, quick, and updated report should be always available covering various information (such as number of infected cases, casualties, regions affected in each country etc.) on easily accessible platforms. The promotion of “Data Science” or Big Data and data driven interdisciplinary research areas has helped a lot to control such global infectious disease or epidemics through extensive surveillance, sharing of epidemiological data, and patient monitoring [144]. Benefits of big data with technological advancement has facilitated the communication among regional, national and international healthcare agencies to monitor future host adaption, viral evolution, infectivity, transmissibility, morbidity, mortality, mental health impact and psychological effects due to COVID-19 epidemic. Here we have mentioned some authentic responsible agencies and tools along with several controversies aroused from the use of data science as follows: 

#### 5.1.1. Responsible Agencies and Devices

**WHO**: WHO is providing a daily “Situation Report” as an update on COVID-19 pandemic (https://www.who.int/emergencies/diseases/novel-coronavirus-2019/situation-reports), where WHO receives the data from national authorities.

**Worldometer**: Worldometer is a free reference website (managed by an international team of developers, researchers, and volunteers) that provides counters and real-time statistics on various topics such as world population, government, economics, society, media, environment, food, water, energy, and health (https://www.worldometers.info/). Now the data in website is currently available in 35 languages and will be available in 11 more languages soon (https://www.worldometers.info/languages/, accessed on 1 May 2020). This reference website belongs to a United States of America (USA)-based digital company Dadax (http://dadax.com/). Presently, this website is being used quite popularly to provide various statistics (graphs, countries, death, symptoms, incubation, transmission, news) on COVID-19 pandemic around the world (https://www.worldometers.info/coronavirus/) and the data is also trusted and used by several agencies including government organization of various countries.

**Mobile phones & COVID-19 Apps:** The speed and ease of communication is the heart of management to control the spread of any infectious diseases as it helps to build extensive surveillance, share of epidemiological data, and patient monitoring. Mobile phone or especially smartphone with the help of various mobile apps (software application) can improve this purpose due to its speedy connectivity, computational power, remote access, electronic reporting, epidemiological data basing, real-time geospatial information, digitized process of contact tracing, more complete and shareable records, enhanced coordination among regional, national and global health agencies [145,146]. A past experience on using smartphones for geospatial tracking of infectious diseases such as HIV, Ebola, and tuberculosis has helped to build these devices further to control COVID-19 epidemics by implementing healthcare strategies, and improving general awareness among masses [147,148,149]. Further, smartphone embedded point-of-care testing and self-reporting facilities has helped to reduce risk of contracting COVID-19 as these help actual and suspected patients with mild symptoms under self-quarantine to report remotely without going to any overcrowded hospital or healthcare centers [150,151]. WHO launched mobile app (https://github.com/WorldHealthOrganization/app) to accurately track and trace COVID-19 cases. There are also several official contact tracing apps available for the citizen of various countries to inform a person’s own health to help governments in strategic control (e.g., “Aarogya Setu” in India, “Covid Watch” in USA, “NHS COVID-19” in UK, "Alipay Health Code" in China, etc.,).

#### 5.1.2. Duel in Data

Despite huge effort and help from data science, dueling in data caused also deviation, divisive and neglecting attitude regarding COVID-19. This further facilitates the spread of pandemic, shifting epicenters, more casualties, economic loss and so many other secondary problems created due to pandemic. Here we have summarized some problems associated with data handling.

**Reproductive number:** The basic reproduction number (R_0_), an indication of the transmissibility of a virus in infectious disease epidemiology, has been used to represent the average number of new COVID-19 infections generated by an infectious person in a totally naïve population [152]. It is estimated that (i) the number of infections is likely to increase for R_0_ > 1, and (ii) transmission is likely to die out for R_0_ < 1. Thus, a true estimation of R_0_ can be beneficial in terms of prevention of any infectious disease. Liu et al. estimated higher reproductive number of COVID-19 compared to SARS coronavirus and also reported that the average reproductive number for COVID-19 (R_0_ ~ 3.28) is considerably higher than that of WHO estimate (R_0_ = 1.95) [153]. They mentioned that the current estimates of R_0_ for COVID-19 are possibly biased due to insufficient data and short onset time.

**“Risk of death” measurement:** Estimation of the case fatality risk or the risk of death among positive cases is a common epidemiological practice to measure the severity of infection for a given disease. Kobayashi et al. [154] mentioned several key epidemiological problems for assessment of the severity of COVID-19 as follows—(i) “division of the cumulative number of deaths by that of cases tends to underestimate the actual risk because deaths that will occur have not yet observed, and so the delay in time from illness onset to death must be addressed”; (ii) “the observed dataset of reported cases represents only a proportion of all infected individuals and there can be a substantial number of asymptomatic and mildly infected individuals who are never diagnosed”; (iii) “ascertainment bias and risk of death among all those infected would be smaller when estimated using shorter virus detection windows and less sensitive diagnostic laboratory tests”. They further suspected that the total number of reported COVID-19 infections will be underestimated due to many undetected mild or asymptomatic cases that go.

**Open data source:** In this fight against COVID-19, an open data source is a big step forward in the age of Big Data. Amaro et al. [155] urged to make an effort through a “Community Letter” for sharing bimolecular simulation data on COVID-19 to help and improve connection and communication among scientists and investigators working on simulation, experimental and clinical data. They committed to share methods, models, and results of their study openly and quickly to test findings, ensure reproducibility, test significance, eliminate dead-ends, and accelerate discovery on COVID-19 applications. They committed to use preprint servers such as arXiv, bioRxiv, and ChemRxiv, open access data repositories such as Zenodo, an open data sharing platforms for models and trajectories such as the Molecular Sciences Software Institute (MolSSI) SARS-CoV-2 Biomolecular Simulation Data and Algorithm Store, the Open Science Framework, the European Open Science Cloud and several other agencies. They also make an appeal and encourage others for similar best practices on open data efforts in other research areas to prevent COVID-19.

**Cyber-attack:** In this grave situation due to COVID-19, the revolutionized advancement of information and communications technology is a crucial weapon to control the healthcare infrastructure in addition to businesses, deliveries of food and essential items to remote places, online grocery shopping etc. But there is a growing concern due to an increasing report of malicious attacks on information and communications technology during COVID-19 as attackers find this an opportunity for financial gains and promoting evil intents. A 220 times increase in spam email and 260% in malicious URLs have been reported from February to March 2020, in which the United States is found to be the epicenter for such targets [156]. Healthcare systems, government and media outlets, financial services are some of most important organization and industries, which are identified at the risk of cyber-attack [157]. This team has identified the top ten such deadly cybersecurity threats amid COVID-19 pandemic, which are as follows—(i) distributed denial of services (DDoS) attack, (ii) malicious domains, (iii) malicious websites, (iv) malware, (v) ransomware, (vi) spam emails, (vii) malicious social media messaging, (viii) business email compromise, (ix) mobile threats, and (x) browsing apps. The Worldometer website was reported under cyber-attack in March 2020 and was then hacked a few days later, resulting in incorrect information (dramatic rise on COVID-19 statistics in Vatican City) for approximately 20 min (https://www.euroweeklynews.com/2020/03/16/false-report-of-900k-dead-in-vatican-city-last-night-i-nearly-fell-off-my-chair-reading-it/). Use of contact tracing COVID-19 apps also raised some security and privacy concern as it keeps personal database of any individual [158,159]. Thus there is a continuous debate on this issue and demand of more reliable apps among mass population, whereas governments are trying to make it a mandatory use as experts shown their confidence about these apps [160].

**Social media activism:** Containment measures are primary guideline to tackle the novel coronavirus (COVID-19) pandemic. Due to moving out of physical public spaces, people are using online platforms as even more prominent and powerful tools to communicate social discussion and understand the unprecedented global crisis. However, Ferrara reported after tracking and studying 43.3M real-time English tweets about COVID-19 (the large-scale data was collected since January 21, 2020, the day the first COVID-19 case was reported on US soil to the dataset up to 12 March 2020, the day before the United States government announced the state of national emergency due to the COVID-19 pandemic) that the information on social media platforms are populated by bots, automated accounts [161]. This study provided the evidence that high bot score accounts are used to amplify certain topics of discussion at the expense of others, such as (i) promotion of political conspiracies and divisive hashtags alongside with COVID-19 content, and (ii) enabling participatory activism to shed light on issues that may otherwise be censored in China. Thus, this study demands more nuanced and regulated discussion on social media platforms on COVID-19.

### 5.2. Precautionary Measurements

Behavioral changes and use of protective gears have been prescribed as the first and foremost precautionary step to stop the spread of SARS-CoV-2. Gradual understanding of the transmission of this virus and previous experience on successful behavioral imposition to control other epidemics such as AIDS (changes in sexual behavior, condom promotion, and government interventions) has helped a lot in that direction. Though more efficient behavioral changes in daily lifestyle requires better understanding and proper implementation of the rules with time on COVID-19 transmission. Awareness on the risks from exposure level to respiratory droplets, airborne virus, contamination level from surfaces, concentration of transmission, incubation period, infectivity even before onset of symptoms in the incubation period, transmission from asymptomatic people will definitely be helpful to understand (i) behavioral changes and precautionary guidelines and (ii) use of protective gears. Further use of telemedicine and robot can also play a crucial role as precautionary steps to combat against such infectious diseases.

**Behavioral changes and precautionary guidelines:** Knowledge on COVID-19 has helped to set precautionary guidelines in our day to day lifestyle. Various health agencies such as WHO, The Centers for Disease Control and Prevention (CDC), National Public Health Institute of the United States and many more recommended following general measures to prevent the spread of COVID-19—(i) complete washing of hands often using an alcohol-based hand sanitizer to kill the SARS-CoV-2, (ii) avoid close contact, (iii) cover mouth and nose with a cloth or mask (e.g., N95 respirators) in public places, (iv) cover coughs and sneezes, (ii) avoid touching eyes, nose and mouth when outside, (iii) avoid travelling or gathering in crowded places, (iv) clean and disinfect frequently touched surfaces, (v) women with infants are encouraged to breastfeed their babies to enhance their immunity (https://www.cdc.gov/coronavirus/2019-ncov/prevent-getting-sick/prevention.html, https://apps.who.int/iris/handle/10665/330376). In addition to hand hygiene practices, WHO also has given proper guidelines for 1. sanitation and plumbing of COVID-19 hospitals, quarantine centers; 2. toilets and the handling of feces of COVID-19 patients; 3. safe management of health care waste; 4. environmental cleaning and laundry at healthcare facilities; 5. safe disposal of greywater or water from washing personal protective gears, surfaces and floors; 6. safe management of dead bodies; 7. management of waste generated at home; 8. treatment and handling requirements for excreta (https://www.who.int/publications-detail/water-sanitation-hygiene-and-waste-management-for-the-covid-19-virus-interim-guidance). The guidance from National Center for Complementary and Integrative Health (NCCIH), National Institutes of Health prescribed a ‘‘healthy waiting’’ in life style, which includes social distancing (or physical distancing), mild exercise, stress reduction, restriction on smoking and alcohol (https://www.nccih.nih.gov/health/in-the-news-coronavirus-and-alternative-treatments). To prevent such health crisis and boost immunity with special reference to respiratory health, Ministry of AYUSH, India recommended the following self-care guidelines in daily lifestyle (https://www.mohfw.gov.in/pdf/ImmunityBoostingAYUSHAdvisory.pdf):

Further, home isolation or quarantine of suspected cases and those with mild illnesses have been entailed as prevention to reduce the burden on COVID-19 hospitals for severe cases [162]. As a preventive measurement, countries have imposed not only inter nation lockdown, but also total or partial lockdown inside their territory to minimize transmission from foreign nationals, social gathering and day to day activity [163]. Lockdown area are also classified based on infection progression and hotspot are also indicated to inform mass about the containment zone. Thermal screening has become a common strategy to track the probable symptomatic cases at various juncture of transportation.

It is obvious that such changes in daily life of general population and healthcare unit need more time to cope with. Implementation of such changes is challenging in short time against rapid infectious nature of COVID-19. According to the psychiatrists and allied professionals, COVID-19 pandemic and such forceful precautionary guidelines created subsyndromal mental health at multiple levels in the general population, among healthcare workers, and in vulnerable populations resulting the symptoms of anxiety and depression, self-reported stress, insufficient sleep [164]. Also, it is surely very conflicting to maintain balance between economy and public health during such long lockdown [165]. Every section of society specially those belonging to lower socio-economic state is being affected economically sooner or later due to such crisis, which create a desperation to neglect such imposed rules on mass population resulting further increase of COVID-19 cases.

**Protective gears:** Various protective gears are playing an important role to stop the spread pandemic. Frontline healthcare professionals, in addition to healthy mass population are at a huge risk in COVID-19 transmission from patients, suspected cases. As of 12 April 2020, more than 2200 healthcare workers including doctors have been infected (https://health.economictimes.indiatimes.com/news/industry/who-says-over-22000-healthcare-workers-across-52-countries-infected-by-covid-19/75107238). In 2002 during the SARS outbreak, nearly 21% of those virus-affected were healthcare workers [166]. This is an even more warning situation as it is important that the healthcare workers should be protected from infection to ensure uninterrupted medical facility and to prevent virus transmission to other patients. Thus, use of protective gears such as Personal Protective Equipment (PPE) kits, mask (tested N95 respirators), gloves and goggles are important accessories for healthcare professionals, whereas for mass population mask (more specifically N95 mask) is foremost important along with other protective guidelines.

Suddenly, very high demand of such protective gears resulted a huge shortage worldwide, which needs a huge workforce to support healthcare facilities in this crisis. Additive manufacturing (i.e., 3D printing), an eminent technology for medical device preparation, came out as an innovative solution for COVID-19 protective gears production with the help of other embedded technologies such as—(i) antimicrobial polymers and nanoparticles for making PPE, (ii) angiotensin-converting enzyme 2 coated nanoparticles containing respiratory masks, chewing gums and nasal filters (iii) preparation of recyclable decontaminating nanofiber filtered face masks, etc. [167,168]. These ideas will surely be advantageous to prepare more effective protective gears for managing such pandemic, though further research is needed to implement such efforts to market.

**Telemedicine:** With the advancement of telecommunication technology, telemedicine has emerged as medical activity involving a doctor-patient distant interaction through telecommunication, which have been interchangeably known as telehealth (more politically correct term) or online health and e-health (fashionable term) [169]. Such telecommunication-based remote medical health facility is surely advantageous against any infectious disease outbreak and thus it is recommended by various health agencies for COVID-19 as it (i) reduces person-to-person contact by obeying social distancing, (ii) maintain balance in facilities with limited workforce, (iii) evolves as an alternative cost effective health provider in comparison to traditional home visit [169,170,171]. In spite of a promising future and willingness to use telehealth among patients, telemedicine suffers from various disadvantageous due to (i) less scope outside of emergency situations, (ii) clinician unwillingness and low acceptance among patients, (iii) problem with reimbursement, (iv) organization of the healthcare system.

**Figure 6:**
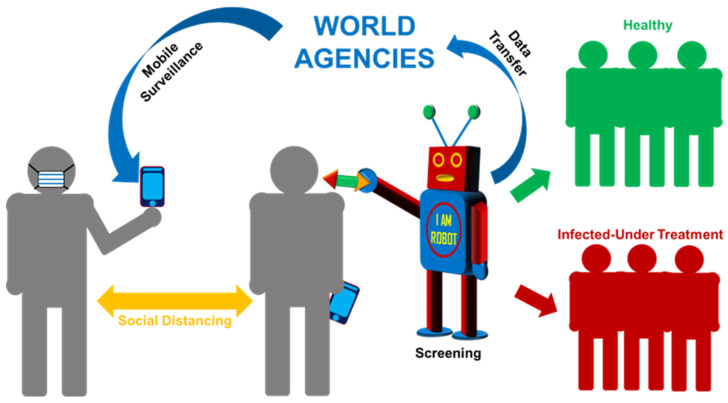
Possible preventive methodologies against infectious disease in future.

**Robotic Gesture:** Robots already shown a huge potential as medical device for diagnosis to surgery including pediatric airway operation, oropharyngeal cancer operation etc. [172,173]. In similar fashion, to combat infectious epidemics like COVID-19, robots can play pivotal roles due to its abilities as follows—(i) large area cleaning and disinfection of contaminated surfaces (such as using UV light devices), (ii) diagnosis by automated or robot-assisted nasopharyngeal and oropharyngeal swab or blood testing, (iii) patient care by delivering medications and food to infected/suspected persons under quarantine, (iv) measuring vital signs, (v) assisting controls on transportation of mass population (such using thermal sensors and vision algorithms), (vi) help in telemedicine, (vii) facial tracking of any individual already having infection as a process of contact tracing, (viii) navigation in high-risk areas for sample transfer as well as delivery of medicines using autonomous drones or robot-assisted ground vehicles [174,175]. Such robot-assisted strategy to control epidemic may speed up the process, reduce the risk of infection specially for frontline health care professionals, and increase workforce (Figure 6). Though despite such help in clinical care, logistics and reconnaissance, proper deployment of robots may face some problem due to privacy and security, incompatible diagnosis with the mutation of virus.

## 6. Conclusions and Future Perspectives

In conclusion, this article presented the current progress of four primary and important sphere of research in the battle against COVID-19 pandemic, which are knowledge of the biological properties of virus, diagnostic modalities, treatment modalities, and prevention modalities. The huge development and deployment of advanced level of research and technology has helped to improve severely affected the global health condition due to COVID-19. A prior knowledge on previous viral outbreaks and pandemic has really helped researchers, health agencies and administrations a lot in response to COVID-19. In spite of such a plethora of research, the progress was hampered in many areas due to conflicting views and controversies about COVID-19. Several such grey areas in transmission, best diagnostic methods with no false positive reports, possible treatment outcome, contact tracing, precautionary steps, proper public health management and many other fields has shattered the progress resulting a chaos and crisis in public health and economy. Despite the conflict, differences in opinions are an important part of any constant progress. Thus, these conflicting views have helped the field to grow and will create unprecedented opportunities further solving several unresolved questions. Governments should encourage and endorse innovative research ideas to beat such pandemics, not only in basic research and biomedicine, but also in engineering, information technology and many unknown corners to get a marketable solution in spite of such controversies. In search of proper treatment, comparatively well-structured diagnosis and prevention are two important armors against COVID-19 with the help of knowledge on biological properties of SARS-CoV-2. We hope that with time, more accurate cumulative knowledge will dissipate the ambiguity about COVID-19 and translational research will help to restore the public life to normalcy. In that aspect, our review would be a huge boost to address the progress amid controversies on a single platform against COVID-19.

## Figures and Tables

**Table 1:** Various detection methods implied for COVID-19 diagnosis.

Test	Method	Sample/Organ
Nucleic acid-based detection	1.Polymerase chain reaction 2. Isothermal nucleic acid amplification 3. CRISPR-Cas	1. Nasal (Nasopharyngeal)/Throat (Oropharyngeal) Swab 2. Blood (Serological test) 3. Fecal swab
Protein-based detection	Viral antibody levels	1. Blood (Serological test) 2. Respiratory swab 3. Fecal
Chest scan (auxiliary test)	1. X-ray 2. Computed tomography	Lung
Autopsy	Surgical (Post-mortem examination)	Corpse

**Table 2:** List of candidate vaccines presently in clinical evaluation for COVID-19 (Source: WHO official website, data accessed on 28 July 2020).

Platform	Type of Vaccine	Developer	Status
Non-Replicating Viral Vector	ChAdOx1-S	University of Oxford/AstraZeneca	Phase 1/2: PACTR202006922165132 2020-001072-15 Phase 2: 2020-001228-32 Phase 3: ISRCTN89951424
Inactivated	Inactivated	Sinovac	Phase 1/2: NCT04383574 NCT04352608 Phase 3: NCT04456595
Inactivated	Inactivated	Wuhan Institute of Biological Products/Sinopharm	Phase 1/2: ChiCTR2000031809 Phase 3: ChiCTR2000034780
Inactivated	Inactivated	Beijing Institute of Biological Products/Sinopharm	Phase 1/2: ChiCTR2000032459 Phase 3: ChiCTR2000034780
RNA	LNP-encapsulated mRNA	Moderna/NIAID	Phase 1: NCT04283461 Phase 2: NCT04405076 Phase 3: NCT04470427
RNA	3 LNP-mRNAs	BioNTech/Fosun Pharma/Pfizer	Phase 1/2: 2020-001038-36 ChiCTR2000034825 Phase 3: NCT04368728
Non-Replicating Viral Vector	Adenovirus Type 5 Vector	CanSino Biological Inc./Beijing Institute of Biotechnology	Phase 1: ChiCTR2000030906 Phase 2: ChiCTR2000031781
Protein Subunit	Adjuvanted recombinant protein (RBD-Dimer)	Anhui Zhifei Longcom Biopharmaceutical/Institute of Microbiology, Chinese Academy of Sciences	Phase 1: NCT04445194 Phase 2: NCT04466085
Inactivated	Inactivated	Institute of Medical Biology, Chinese Academy of Medical Sciences	Phase 1: NCT04412538 Phase 1/2: NCT04470609
DNA	DNA plasmid vaccine with electroporation	Inovio Pharmaceuticals/International Vaccine Institute	Phase 1/2: NCT04447781 NCT04336410
DNA	DNA plasmid vaccine + Adjuvant	Osaka University/AnGes/Takara Bio	Phase 1/2: NCT04463472
DNA	DNA plasmid vaccine	Cadila Healthcare Limited	Phase 1/2: CTRI/2020/07/026352
DNA	DNA Vaccine (GX-19)	Genexine Consortium	Phase 1/2: NCT04445389
Inactivated	Whole-Virion Inactivated	Bharat Biotech	NA
Protein Subunit	Full length recombinant SARS CoV-2 glycoprotein NPs vaccine adjuvanted with Matrix M	Novavax	Phase 1/2: NCT04368988
Protein Subunit	RBD-based	Kentucky Bioprocessing, Inc	Phase 1/2: NCT04473690
RNA	mRNA	Arcturus/Duke-NUS	Phase 1/2: NCT04480957
Non-Replicating Viral Vector	Adeno-based	Gamaleya Research Institute	Phase 1: NCT04436471 NCT04437875
Protein Subunit	Native like Trimeric subunit Spike Protein vaccine	Clover Biopharmaceuticals Inc./GSK/Dynavax	Phase 1: NCT04405908
Protein Subunit	Recombinant spike protein with Advax™ adjuvant	Vaxine Pty Ltd./Medytox	Phase 1: NCT04453852
Protein Subunit	Molecular clamp stabilized Spike protein with MF59 adjuvant	University of Queensland/CSL/Seqirus	Phase 1: ACTRN12620000674932p
RNA	LNP-nCoVsaRNA	Imperial College London	Phase 1: ISRCTN17072692
RNA	mRNA	Curevac	Phase 1: NCT04449276
RNA	mRNA	People’s Liberation Army (PLA) Academy of Military Sciences/Walvax Biotech.	Phase 1: ChiCTR2000034112
VLP	Plant-derived VLP adjuvanted with GSK or Dynavax adjs.	Medicago Inc.	Phase 1: NCT04450004
Protein Subunit	S-2P protein + CpG 1018	Medigen Vaccine Biologics Corporation/NIAID/Dynavax	Phase 1: NCT04487210

## References

[1] McIntosh K. (1974). Coronaviruses: A Comparative Review.

[2] Contini C., Di Nuzzo M., Barp N., Bonazza A., De Giorgio R., Tognon M., Rubino S. (2020). The novel zoonotic COVID-19 pandemic: An expected global health concern. J. Infect. Dev. Ctries..

[3] Schoeman D., Fielding B.C. (2019). Coronavirus envelope protein: Current knowledge. Virol. J..

[4] McIntosh K., Peiris J.S.M. (2009). Coronaviruses. Clinical Virology.

[5] Walls A.C., Park Y.J., Tortorici M.A., Wall A., McGuire A.T., Veesler D. (2020). Structure, Function, and Antigenicity of the SARS-CoV-2 Spike Glycoprotein. Cell.

[6] Andersen K.G., Rambaut A., Lipkin W.I., Holmes E.C., Garry R.F. (2020). The proximal origin of SARS-CoV-2. Nat. Med..

[7] Velavan T.P., Meyer C.G. (2020). The COVID-19 epidemic. Trop. Med. Int. Health TM IH.

[8] Weiss S.R., Navas-Martin S. (2005). Coronavirus pathogenesis and the emerging pathogen severe acute respiratory syndrome coronavirus. Microbiol. Mol. Biol. Rev..

[9] Chan J.F., Yuan S., Kok K.H., To K.K., Chu H., Yang J., Xing F., Liu J., Yip C.C., Poon R.W. (2020). A familial cluster of pneumonia associated with the 2019 novel coronavirus indicating person-to-person transmission: A study of a family cluster. Lancet.

[10] Chan J.F., Kok K.H., Zhu Z., Chu H., To K.K., Yuan S., Yuen K.Y. (2020). Genomic characterization of the 2019 novel human-pathogenic coronavirus isolated from a patient with atypical pneumonia after visiting Wuhan. Emerg. Microbes Infect..

[11] van Doremalen N., Bushmaker T., Morris D.H., Holbrook M.G., Gamble A., Williamson B.N., Tamin A., Harcourt J.L., Thornburg N.J., Gerber S.I. (2020). Aerosol and Surface Stability of SARS-CoV-2 as Compared with SARS-CoV-1. N. Engl. J. Med..

[12] Zhu N., Zhang D., Wang W., Li X., Yang B., Song J., Zhao X., Huang B., Shi W., Lu R. (2020). A Novel Coronavirus from Patients with Pneumonia in China, 2019. N. Engl. J. Med..

[13] Public Health Image Library (PHIL). https://phil.cdc.gov/Details.aspx?pid=23354.

[14] Zhang L., Lin D., Sun X., Curth U., Drosten C., Sauerhering L., Becker S., Rox K., Hilgenfeld R. (2020). Crystal structure of SARS-CoV-2 main protease provides a basis for design of improved α-ketoamide inhibitors. Science.

[15] Zhou P., Fan H., Lan T., Yang X.L., Shi W.F., Zhang W., Zhu Y., Zhang Y.W., Xie Q.M., Mani S. (2018). Fatal swine acute diarrhoea syndrome caused by an HKU2-related coronavirus of bat origin. Nature.

[16] Xu R.H., He J.F., Evans M.R., Peng G.W., Field H.E., Yu D.W., Lee C.K., Luo H.M., Lin W.S., Lin P. (2004). Epidemiologic clues to SARS origin in China. Emerg. Infect. Dis..

[17] Fan Y., Zhao K., Shi Z.L., Zhou P. (2019). Bat Coronaviruses in China. Viruses.

[18] Singhal T. (2020). A Review of Coronavirus Disease-2019 (COVID-19). Indian J. Pediatr..

[19] Zhang T., Wu Q., Zhang Z. (2020). Probable Pangolin Origin of SARS-CoV-2 Associated with the COVID-19 Outbreak. Curr. Biol. CB.

[20] Li X., Zai J., Zhao Q., Nie Q., Li Y., Foley B.T., Chaillon A. (2020). Evolutionary history, potential intermediate animal host, and cross-species analyses of SARS-CoV-2. J. Med. Virol..

[21] https://www.cdc.gov/mmwr/volumes/69/wr/mm6909e1.htm.

[22] Zhou P., Yang X.L., Wang X.G., Hu B., Zhang L., Zhang W., Si H.R., Zhu Y., Li B., Huang C.L. (2020). A pneumonia outbreak associated with a new coronavirus of probable bat origin. Nature.

[23] Holshue M.L., DeBolt C., Lindquist S., Lofy K.H., Wiesman J., Bruce H., Spitters C., Ericson K., Wilkerson S., Tural A. (2020). First Case of 2019 Novel Coronavirus in the United States. N. Engl. J. Med..

[24] Wang Q., Zhang Y., Wu L., Niu S., Song C., Zhang Z., Lu G., Qiao C., Hu Y., Yuen K.Y. (2020). Structural and Functional Basis of SARS-CoV-2 Entry by Using Human ACE2. Cell.

[25] Hamming I., Timens W., Bulthuis M.L., Lely A.T., Navis G., van Goor H. (2004). Tissue distribution of ACE2 protein, the functional receptor for SARS coronavirus. A first step in understanding SARS pathogenesis. J. Pathol..

[26] Udugama B., Kadhiresan P., Kozlowski H.N., Malekjahani A., Osborne M., Li V.Y.C., Chen H., Mubareka S., Gubbay J.B., Chan W.C.W. (2020). Diagnosing COVID-19: The Disease and Tools for Detection. ACS Nano.

[27] Zou L., Ruan F., Huang M., Liang L., Huang H., Hong Z., Yu J., Kang M., Song Y., Xia J. (2020). SARS-CoV-2 Viral Load in Upper Respiratory Specimens of Infected Patients. N. Engl. J. Med..

[28] Mullis K.B. (1990). The unusual origin of the polymerase chain reaction. Sci. Am..

[29] Saiki R.K., Gelfand D.H., Stoffel S., Scharf S.J., Higuchi R., Horn G.T., Mullis K.B., Erlich H.A. (1988). Primer-directed enzymatic amplification of DNA with a thermostable DNA polymerase. Science.

[30] Adachi D., Johnson G., Draker R., Ayers M., Mazzulli T., Talbot P.J., Tellier R. (2004). Comprehensive detection and identification of human coronaviruses, including the SARS-associated coronavirus, with a single RT-PCR assay. J. Virol. Methods.

[31] Setianingsih T.Y., Wiyatno A., Hartono T.S., Hindawati E., Dewantari A.K., Myint K.S., Lisdawati V., Safari D. (2019). Detection of multiple viral sequences in the respiratory tract samples of suspected Middle East respiratory syndrome coronavirus patients in Jakarta, Indonesia 2015–2016. Int. J. Infect. Dis. Ijid Off. Publ. Int. Soc. Infect. Dis..

[32] Corman V.M., Eckerle I., Bleicker T., Zaki A., Landt O., Eschbach-Bludau M., van Boheemen S., Gopal R., Ballhause M., Bestebroer T.M. (2012). Detection of a novel human coronavirus by real-time reverse-transcription polymerase chain reaction. Euro Surveill. Bull. Eur. Sur Les Mal. Transm. Eur. Commun. Dis. Bull..

[33] Zhai J., Briese T., Dai E., Wang X., Pang X., Du Z., Liu H., Wang J., Wang H., Guo Z. (2004). Real-time polymerase chain reaction for detecting SARS coronavirus, Beijing, 2003. Emerg. Infect. Dis..

[34] Corman V.M., Landt O., Kaiser M., Molenkamp R., Meijer A., Chu D.K., Bleicker T., Brünink S., Schneider J., Schmidt M.L. (2020). Detection of 2019 novel coronavirus (2019-nCoV) by real-time RT-PCR. Euro Surveill. Bull. Eur. Sur Les Mal. Transm. Eur. Commun. Dis. Bull..

[35] Konrad R., Eberle U., Dangel A., Treis B., Berger A., Bengs K., Fingerle V., Liebl B., Ackermann N., Sing A. (2020). Rapid establishment of laboratory diagnostics for the novel coronavirus SARS-CoV-2 in Bavaria, Germany, February 2020. Eurosurveillance.

[36] Xu Y., Li X., Zhu B., Liang H., Fang C., Gong Y., Guo Q., Sun X., Zhao D., Shen J. (2020). Characteristics of pediatric SARS-CoV-2 infection and potential evidence for persistent fecal viral shedding. Nat. Med..

[37] Pan Y., Zhang D., Yang P., Poon L.L.M., Wang Q. (2020). Viral load of SARS-CoV-2 in clinical samples. Lancet. Infect. Dis..

[38] Chan J.F.-W., Yip C.C.-Y., To K.K.-W., Tang T.H.-C., Wong S.C.-Y., Leung K.-H., Fung A.Y.-F., Ng A.C.-K., Zou Z., Tsoi H.-W. (2020). Improved Molecular Diagnosis of COVID-19 by the Novel, Highly Sensitive and Specific COVID-19-RdRp/Hel Real-Time Reverse Transcription-PCR Assay Validated In Vitro and with Clinical Specimens. J. Clin. Microbiol..

[39] Pfefferle S., Reucher S., Nörz D., Lütgehetmann M. (2020). Evaluation of a quantitative RT-PCR assay for the detection of the emerging coronavirus SARS-CoV-2 using a high throughput system. Euro Surveill. Bull. Eur. Sur Les Mal. Transm. Eur. Commun. Dis. Bull..

[40] Zhao Y., Chen F., Li Q., Wang L., Fan C. (2015). Isothermal Amplification of Nucleic Acids. Chem. Rev..

[41] Gill P., Ghaemi A. (2008). Nucleic acid isothermal amplification technologies: A review. Nucleosides Nucleotides Nucleic Acids.

[42] Shen M., Zhou Y., Ye J., Abdullah Al-Maskri A.A., Kang Y., Zeng S., Cai S. (2020). Recent advances and perspectives of nucleic acid detection for coronavirus. J. Pharm. Anal..

[43] Poon L.L., Leung C.S., Tashiro M., Chan K.H., Wong B.W., Yuen K.Y., Guan Y., Peiris J.S. (2004). Rapid detection of the severe acute respiratory syndrome (SARS) coronavirus by a loop-mediated isothermal amplification assay. Clin. Chem..

[44] Shirato K., Semba S., El-Kafrawy S.A., Hassan A.M., Tolah A.M., Takayama I., Kageyama T., Notomi T., Kamitani W., Matsuyama S. (2018). Development of fluorescent reverse transcription loop-mediated isothermal amplification (RT-LAMP) using quenching probes for the detection of the Middle East respiratory syndrome coronavirus. J. Virol. Methods.

[45] Wang W.K., Fang C.T., Chen H.L., Yang C.F., Chen Y.C., Chen M.L., Chen S.Y., Yang J.Y., Lin J.H., Yang P.C. (2005). Detection of severe acute respiratory syndrome coronavirus RNA in plasma during the course of infection. J. Clin. Microbiol..

[46] Park G.S., Ku K., Baek S.H., Kim S.J., Kim S.I., Kim B.T., Maeng J.S. (2020). Development of Reverse Transcription Loop-Mediated Isothermal Amplification Assays Targeting Severe Acute Respiratory Syndrome Coronavirus 2 (SARS-CoV-2). J. Mol. Diagn. JMD.

[47] Zhu X., Wang X., Han L., Chen T., Wang L., Li H., Li S., He L., Fu X., Chen S. (2020). Reverse transcription loop-mediated isothermal amplification combined with nanoparticles-based biosensor for diagnosis of COVID-19. medRxiv.

[48] Zhang Y., Odiwuor N., Xiong J., Sun L., Nyaruaba R.O., Wei H., Tanner N. (2020). Rapid Molecular Detection of SARS-CoV-2 (COVID-19) Virus RNA Using Colorimetric LAMP. medRxiv.

[49] Yu L., Wu S., Hao X., Dong X., Mao L., Pelechano V., Chen W.H., Yin X. (2020). Rapid detection of COVID-19 coronavirus using a reverse transcriptional loop-mediated isothermal amplification (RT-LAMP) diagnostic platform. Clin. Chem..

[50] Makarova K.S., Wolf Y.I., Alkhnbashi O.S., Costa F., Shah S.A., Saunders S.J., Barrangou R., Brouns S.J., Charpentier E., Haft D.H. (2015). An updated evolutionary classification of CRISPR-Cas systems. Nat. Rev. Microbiol..

[51] Wright A.V., Nuñez J.K., Doudna J.A. (2016). Biology and Applications of CRISPR Systems: Harnessing Nature’s Toolbox for Genome Engineering. Cell.

[52] Pickar-Oliver A., Gersbach C.A. (2019). The next generation of CRISPR-Cas technologies and applications. Nat. Rev. Mol. Cell Biol..

[53] Gootenberg J.S., Abudayyeh O.O., Lee J.W., Essletzbichler P., Dy A.J., Joung J., Verdine V., Donghia N., Daringer N.M., Freije C.A. (2017). Nucleic acid detection with CRISPR-Cas13a/C2c2. Science.

[54] Freije C.A., Myhrvold C., Boehm C.K., Lin A.E., Welch N.L., Carter A., Metsky H.C., Luo C.Y., Abudayyeh O.O., Gootenberg J.S. (2019). Programmable Inhibition and Detection of RNA Viruses Using Cas13. Mol. Cell.

[55] Gootenberg J.S., Abudayyeh O.O., Kellner M.J., Joung J., Collins J.J., Zhang F. (2018). Multiplexed and portable nucleic acid detection platform with Cas13, Cas12a, and Csm6. Science.

[56] Broughton J.P., Deng X., Yu G., Fasching C.L., Servellita V., Singh J., Miao X., Streithorst J.A., Granados A., Sotomayor-Gonzalez A. (2020). CRISPR-Cas12-based detection of SARS-CoV-2. Nat. Biotechnol..

[57] Azhar M., Phutela R., Ansari A.H., Sinha D., Sharma N., Kumar M., Aich M., Sharma S., Rauthan R., Singhal K. (2020). Rapid, field-deployable nucleobase detection and identification using FnCas9. bioRxiv.

[58] Kaye H.S., Ong S.B., Dowdle W.R. (1972). Detection of coronavirus 229E antibody by indirect hemagglutination. Appl. Microbiol..

[59] Hu Z., Song C., Xu C., Jin G., Chen Y., Xu X., Ma H., Chen W., Lin Y., Zheng Y. (2020). Clinical characteristics of 24 asymptomatic infections with COVID-19 screened among close contacts in Nanjing, China. Sci. China. Life Sci..

[60] Li Z., Yi Y., Luo X., Xiong N., Liu Y., Li S., Sun R., Wang Y., Hu B., Chen W. (2020). Development and clinical application of a rapid IgM-IgG combined antibody test for SARS-CoV-2 infection diagnosis. J. Med. Virol..

[61] To K.K., Tsang O.T., Leung W.S., Tam A.R., Wu T.C., Lung D.C., Yip C.C., Cai J.P., Chan J.M., Chik T.S. (2020). Temporal profiles of viral load in posterior oropharyngeal saliva samples and serum antibody responses during infection by SARS-CoV-2: An observational cohort study. Lancet. Infect. Dis..

[62] Zhang W., Du R.H., Li B., Zheng X.S., Yang X.L., Hu B., Wang Y.Y., Xiao G.F., Yan B., Shi Z.L. (2020). Molecular and serological investigation of 2019-nCoV infected patients: Implication of multiple shedding routes. Emerg. Microbes Infect..

[63] Guo L., Ren L., Yang S., Xiao M., Chang D., Yang F., Dela Cruz C.S., Wang Y., Wu C., Xiao Y. (2020). Profiling Early Humoral Response to Diagnose Novel Coronavirus Disease (COVID-19). Clin. Infect. Dis. Off. Publ. Infect. Dis. Soc. Am..

[64] Seo G., Lee G., Kim M.J., Baek S.H., Choi M., Ku K.B., Lee C.S., Jun S., Park D., Kim H.G. (2020). Rapid Detection of COVID-19 Causative Virus (SARS-CoV-2) in Human Nasopharyngeal Swab Specimens Using Field-Effect Transistor-Based Biosensor. ACS Nano.

[65] Lv H., Wu N.C., Tsang O.T.-Y., Yuan M., Perera R.A.P.M., Leung W.S., So R.T.Y., Chan J.M.C., Yip G.K., Chik T.S.H. (2020). Cross-reactive Antibody Response between SARS-CoV-2 and SARS-CoV Infections. Cell Rep..

[66] Guan W.J., Ni Z.Y., Hu Y., Liang W.H., Ou C.Q., He J.X., Liu L., Shan H., Lei C.L., Hui D.S.C. (2020). Clinical Characteristics of Coronavirus Disease 2019 in China. N. Engl. J. Med..

[67] Hayden G.E., Wrenn K.W. (2009). Chest radiograph vs. computed tomography scan in the evaluation for pneumonia. J. Emerg. Med..

[68] Hamimi A. (2016). MERS-CoV: Middle East respiratory syndrome corona virus: Can radiology be of help? Initial single center experience. Egypt. J. Radiol. Nucl. Med..

[69] Xie X., Li X., Wan S., Gong Y., Williams G.J., Simoff S.J. (2006). Mining X-Ray Images of SARS Patients. Data Mining: Theory, Methodology, Techniques, and Applications.

[70] Zhang W. (2020). Imaging changes of severe COVID-19 pneumonia in advanced stage. Intensive Care Med..

[71] Ozturk T., Talo M., Yildirim E.A., Baloglu U.B., Yildirim O., Rajendra Acharya U. (2020). Automated detection of COVID-19 cases using deep neural networks with X-ray images. Comput. Biol. Med..

[72] Sobue T., Moriyama N., Kaneko M., Kusumoto M., Kobayashi T., Tsuchiya R., Kakinuma R., Ohmatsu H., Nagai K., Nishiyama H. (2002). Screening for lung cancer with low-dose helical computed tomography: Anti-lung cancer association project. J. Clin. Oncol. Off. J. Am. Soc. Clin. Oncol..

[73] Claessens Y.E., Debray M.P., Tubach F., Brun A.L., Rammaert B., Hausfater P., Naccache J.M., Ray P., Choquet C., Carette M.F. (2015). Early Chest Computed Tomography Scan to Assist Diagnosis and Guide Treatment Decision for Suspected Community-acquired Pneumonia. Am. J. Respir. Crit. Care Med..

[74] Yang W., Yan F. (2020). Patients with RT-PCR-confirmed COVID-19 and Normal Chest CT. Radiology.

[75] Fang Y., Zhang H., Xie J., Lin M., Ying L., Pang P., Ji W. (2020). Sensitivity of Chest CT for COVID-19: Comparison to RT-PCR. Radiology.

[76] Ai T., Yang Z., Hou H., Zhan C., Chen C., Lv W., Tao Q., Sun Z., Xia L. (2020). Correlation of Chest CT and RT-PCR Testing in Coronavirus Disease 2019 (COVID-19) in China: A Report of 1014 Cases. Radiology.

[77] Chung M., Bernheim A., Mei X., Zhang N., Huang M., Zeng X., Cui J., Xu W., Yang Y., Fayad Z.A. (2020). CT Imaging Features of 2019 Novel Coronavirus (2019-nCoV). Radiology.

[78] Shi H., Han X., Zheng C. (2020). Evolution of CT Manifestations in a Patient Recovered from 2019 Novel Coronavirus (2019-nCoV) Pneumonia in Wuhan, China. Radiology.

[79] Pan Y., Guan H., Zhou S., Wang Y., Li Q., Zhu T., Hu Q., Xia L. (2020). Initial CT findings and temporal changes in patients with the novel coronavirus pneumonia (2019-nCoV): A study of 63 patients in Wuhan, China. Eur. Radiol..

[80] Gozes O., Frid-Adar M., Greenspan H., Browning P.D., Zhang H., Ji W., Bernheim A., Siegel E. (2020). Rapid AI Development Cycle for the Coronavirus (COVID-19) Pandemic: Initial Results for Automated Detection & Patient Monitoring using Deep Learning CT Image Analysis. arXiv.

[81] Shan F., Gao Y., Wang J., Shi W., Shi N., Han M., Xue Z., Shen D., Shi Y. (2020). Lung Infection Quantification of COVID-19 in CT Images with Deep Learning. arXiv.

[82] Butt C., Gill J., Chun D., Babu B.A. (2020). Deep learning system to screen coronavirus disease 2019 pneumonia. Appl. Intell..

[83] Wang S., Kang B., Ma J., Zeng X., Xiao M., Guo J., Cai M., Yang J., Li Y., Meng X. (2020). A deep learning algorithm using CT images to screen for Corona Virus Disease (COVID-19). medRxiv.

[84] Schwartz D.A., Herman C.J. (1996). The importance of the autopsy in emerging and reemerging infectious diseases. Clin. Infect. Dis. Off. Publ. Infect. Dis. Soc. Am..

[85] Salerno M., Sessa F., Piscopo A., Montana A., Torrisi M., Patane F., Murabito P., Volti G.L., Pomara C. (2020). No Autopsies on COVID-19 Deaths: A Missed Opportunity and the Lockdown of Science. J. Clin. Med..

[86] Hanley B., Lucas S.B., Youd E., Swift B., Osborn M. (2020). Autopsy in suspected COVID-19 cases. J. Clin. Pathol..

[87] Pomara C., Li Volti G., Cappello F. (2020). COVID-19 Deaths: Are We Sure It Is Pneumonia? Please, Autopsy, Autopsy, Autopsy!. J. Clin. Med..

[88] http://www.trovanorme.salute.gov.it/norme/renderNormsanPdf?anno=2020&codLeg=73965&parte=1%20&serie=null.

[89] Sapino A., Facchetti F., Bonoldi E., Gianatti A., Barbareschi M., Societa Italiana di Anatomia Patologica e Citologia-SIAPEC (2020). The autopsy debate during the COVID-19 emergency: The Italian experience. Virchows Arch..

[90] Chang C.K., Lo S.C., Wang Y.S., Hou M.H. (2016). Recent insights into the development of therapeutics against coronavirus diseases by targeting N protein. Drug Discov. Today.

[91] Li X., Geng M., Peng Y., Meng L., Lu S. (2020). Molecular immune pathogenesis and diagnosis of COVID-19. J. Pharm. Anal..

[92] De Clercq E., Li G. (2016). Approved Antiviral Drugs over the Past 50 Years. Clin. Microbiol. Rev..

[93] Senanayake S.L. (2020). Drug repurposing strategies for COVID-19. Future Drug Discov.

[94] Pushpakom S., Iorio F., Eyers P.A., Escott K.J., Hopper S., Wells A., Doig A., Guilliams T., Latimer J., McNamee C. (2019). Drug repurposing: Progress, challenges and recommendations. Nat. Rev. Drug Discov..

[95] Li C.C., Wang X.J., Wang H.R. (2019). Repurposing host-based therapeutics to control coronavirus and influenza virus. Drug Discov. Today.

[96] Hodos R.A., Kidd B.A., Shameer K., Readhead B.P., Dudley J.T. (2016). In silico methods for drug repurposing and pharmacology. Wiley Interdiscip. Rev. Syst. Biol. Med..

[97] Agamah F.E., Mazandu G.K., Hassan R., Bope C.D., Thomford N.E., Ghansah A., Chimusa E.R. (2019). Computational/in silico methods in drug target and lead prediction. Brief. Bioinform..

[98] Pawar A.Y. (2020). Combating Devastating COVID -19 by Drug Repurposing. Int. J. Antimicrob. Agents.

[99] Phadke M., Saunik S. (2020). COVID-19 treatment by repurposing drugs until the vaccine is in sight. Drug Dev. Res..

[100] Wang J. (2020). Fast Identification of Possible Drug Treatment of Coronavirus Disease-19 (COVID-19) through Computational Drug Repurposing Study. J. Chem. Inf. Modeling.

[101] Ciliberto G., Cardone L. (2020). Boosting the arsenal against COVID-19 through computational drug repurposing. Drug Discov. Today.

[102] Kupferschmidt K., Cohen J. (2020). WHO launches global megatrial of the four most promising coronavirus treatments. Science.

[103] Cheng M.P., Lee T.C., Tan D.H.S., Murthy S. (2020). Generating randomized trial evidence to optimize treatment in the COVID-19 pandemic. CMAJ Can. Med Assoc. J. J. De L’association Med. Can..

[104] Belhadi D., Peiffer-Smadja N., Lescure F.-X., Yazdanpanah Y., Mentré F., Laouénan C. (2020). A brief review of antiviral drugs evaluated in registered clinical trials for COVID-19. medRxiv.

[105] Wang Y., Zhang D., Du G., Du R., Zhao J., Jin Y., Fu S., Gao L., Cheng Z., Lu Q. (2020). Remdesivir in adults with severe COVID-19: A randomised, double-blind, placebo-controlled, multicentre trial. Lancet.

[106] Gautret P., Lagier J.C., Parola P., Hoang V.T., Meddeb L., Mailhe M., Doudier B., Courjon J., Giordanengo V., Vieira V.E. (2020). Hydroxychloroquine and azithromycin as a treatment of COVID-19: Results of an open-label non-randomized clinical trial. Int. J. Antimicrob. Agents.

[107] Yazdany J., Kim A.H.J. (2020). Use of Hydroxychloroquine and Chloroquine During the COVID-19 Pandemic: What Every Clinician Should Know. Ann. Intern. Med..

[108] Cao B., Wang Y., Wen D., Liu W., Wang J., Fan G., Ruan L., Song B., Cai Y., Wei M. (2020). A Trial of Lopinavir-Ritonavir in Adults Hospitalized with Severe Covid-19. N. Engl. J. Med..

[109] Naz R.K., Gupta S.K., Gupta J.C., Vyas H.K., Talwar A.G. (2005). Recent advances in contraceptive vaccine development: A mini-review. Hum. Reprod..

[110] Girard M.P., Osmanov S.K., Kieny M.P. (2006). A review of vaccine research and development: The human immunodeficiency virus (HIV). Vaccine.

[111] Liu C., Zhou Q., Li Y., Garner L.V., Watkins S.P., Carter L.J., Smoot J., Gregg A.C., Daniels A.D., Jervey S. (2020). Research and Development on Therapeutic Agents and Vaccines for COVID-19 and Related Human Coronavirus Diseases. ACS Cent Sci..

[112] Saif L.J. Vaccines for COVID-19: Perspectives, Prospects, and Challenges Based on Candidate SARS, MERS, and Animal Coronavirus Vaccines. EMJ.

[113] Du L., He Y., Zhou Y., Liu S., Zheng B.-J., Jiang S. (2009). The spike protein of SARS-CoV--a target for vaccine and therapeutic development. Nat. Rev. Microbiol..

[114] Thanh Le T., Andreadakis Z., Kumar A., Gómez Román R., Tollefsen S., Saville M., Mayhew S. (2020). The COVID-19 vaccine development landscape. Nat. Rev. Drug Discov..

[115] Chen W.H., Strych U., Hotez P.J., Bottazzi M.E. (2020). The SARS-CoV-2 Vaccine Pipeline: An Overview. Curr. Trop. Med. Rep..

[116] Peeples L. (2020). News Feature: Avoiding pitfalls in the pursuit of a COVID-19 vaccine. Proc. Natl. Acad. Sci. USA.

[117] Yamey G., Schäferhoff M., Hatchett R., Pate M., Zhao F., McDade K.K. (2020). Ensuring global access to COVID-19 vaccines. Lancet.

[118] Lucchese G., Flöel A. (2020). Molecular mimicry between SARS-CoV-2 and respiratory pacemaker neurons. Autoimmun. Rev..

[119] Angileri F., Legare S., Marino Gammazza A., Conway de Macario E., Jl Macario A., Cappello F. (2020). Molecular mimicry may explain multi-organ damage in COVID-19. Autoimmun. Rev..

[120] Angileri F., Légaré S., Marino Gammazza A., Conway de Macario E., Macario A.J.L., Cappello F. (2020). Is molecular mimicry the culprit in the autoimmune haemolytic anaemia affecting patients with COVID-19?. Br. J. Haematol..

[121] Cegolon L., Pichierri J., Mastrangelo G., Cinquetti S., Sotgiu G., Bellizzi S., Pichierri G. (2020). Hypothesis to explain the severe form of COVID-19 in Northern Italy. BMJ Glob. Health.

[122] Marano G., Vaglio S., Pupella S., Facco G., Catalano L., Liumbruno G.M., Grazzini G. (2016). Convalescent plasma: New evidence for an old therapeutic tool?. Blood Transfus.

[123] Cheng Y., Wong R., Soo Y.O., Wong W.S., Lee C.K., Ng M.H., Chan P., Wong K.C., Leung C.B., Cheng G. (2005). Use of convalescent plasma therapy in SARS patients in Hong Kong. Eur. J. Clin. Microbiol. Infect. Dis. Off. Publ. Eur. Soc. Clin. Microbiol..

[124] Zhou B., Zhong N., Guan Y. (2007). Treatment with convalescent plasma for influenza A (H5N1) infection. N. Engl. J. Med..

[125] Cunningham A.C., Goh H.P., Koh D. (2020). Treatment of COVID-19: Old tricks for new challenges. Crit. Care.

[126] Duan K., Liu B., Li C., Zhang H., Yu T., Qu J., Zhou M., Chen L., Meng S., Hu Y. (2020). Effectiveness of convalescent plasma therapy in severe COVID-19 patients. Proc. Natl. Acad. Sci. USA.

[127] Menis M., Sridhar G., Selvam N., Ovanesov M.V., Divan H.A., Liang Y., Scott D., Golding B., Forshee R., Ball R. (2013). Hyperimmune globulins and same-day thrombotic adverse events as recorded in a large healthcare database during 2008-2011. Am. J. Hematol..

[128] Casadevall A., Pirofski L.A. (2020). The convalescent sera option for containing COVID-19. J. Clin. Investig..

[129] Wang L., Wang Y., Ye D., Liu Q. (2020). Review of the 2019 novel coronavirus (SARS-CoV-2) based on current evidence. Int. J. Antimicrob. Agents.

[130] Lim C.C., Tan C.S., Kaushik M., Tan H.K. (2015). Initiating acute dialysis at earlier Acute Kidney Injury Network stage in critically ill patients without traditional indications does not improve outcome: A prospective cohort study. Nephrology.

[131] Zarbock A., Kellum J.A., Schmidt C., Van Aken H., Wempe C., Pavenstädt H., Boanta A., Gerß J., Meersch M. (2016). Effect of Early vs Delayed Initiation of Renal Replacement Therapy on Mortality in Critically Ill Patients With Acute Kidney Injury: The ELAIN Randomized Clinical Trial. JAMA.

[132] Wang S.-X., Wang Y., Lu Y.-B., Li J.-Y., Song Y.-J., Nyamgerelt M., Wang X.-X. (2020). Diagnosis and treatment of novel coronavirus pneumonia based on the theory of traditional Chinese medicine. J. Integr. Med..

[133] Bodeker G., Kronenberg F. (2002). A public health agenda for traditional, complementary, and alternative medicine. Am. J. Public Health.

[134] Ernst E. (2000). The role of complementary and alternative medicine. BMJ.

[135] Maurya V.K., Kumar S., Bhatt M.L.B., Saxena S.K. (2020). Therapeutic Development and Drugs for the Treatment of COVID-19. Coronavirus Disease 2019 (COVID-19).

[136] Saxena S.K., Haikerwal A., Gadugu S., Bhatt M.L. (2017). Complementary and alternative medicine in alliance with conventional medicine for dengue therapeutics and prevention. Future Med..

[137] Ravishankar B., Shukla V.J. (2007). Indian systems of medicine: A brief profile. Afr. J. Tradit. Complement. Altern. Med..

[138] Vellingiri B., Jayaramayya K., Iyer M., Narayanasamy A., Govindasamy V., Giridharan B., Ganesan S., Venugopal A., Venkatesan D., Ganesan H. (2020). COVID-19: A promising cure for the global panic. Sci. Total Environ..

[139] Vimalanathan S., Ignacimuthu S., Hudson J.B. (2009). Medicinal plants of Tamil Nadu (Southern India) are a rich source of antiviral activities. Pharm. Biol..

[140] Yang Y., Islam M.S., Wang J., Li Y., Chen X. (2020). Traditional Chinese Medicine in the Treatment of Patients Infected with 2019-New Coronavirus (SARS-CoV-2): A Review and Perspective. Int. J. Biol. Sci..

[141] Li H., Zhou Y., Zhang M., Wang H., Zhao Q., Liu J. (2020). Updated Approaches against SARS-CoV-2. Antimicrob. Agents Chemother..

[142] Wang Z., Chen X., Lu Y., Chen F., Zhang W. (2020). Clinical characteristics and therapeutic procedure for four cases with 2019 novel coronavirus pneumonia receiving combined Chinese and Western medicine treatment. Biosci. Trends.

[143] Weeks J. (2020). Call to Action: Announcing the Traditional, Complementary and Integrative Health and Medicine COVID-19 Support Registry. J. Altern. Complement. Med..

[144] Hay S.I., George D.B., Moyes C.L., Brownstein J.S. (2013). Big data opportunities for global infectious disease surveillance. PLoS Med..

[145] Wood C.S., Thomas M.R., Budd J., Mashamba-Thompson T.P., Herbst K., Pillay D., Peeling R.W., Johnson A.M., McKendry R.A., Stevens M.M. (2019). Taking connected mobile-health diagnostics of infectious diseases to the field. Nature.

[146] Nayak S., Blumenfeld N.R., Laksanasopin T., Sia S.K. (2017). Point-of-Care Diagnostics: Recent Developments in a Connected Age. Anal. Chem..

[147] Swendeman D., Rotheram-Borus M.J. (2010). Innovation in sexually transmitted disease and HIV prevention: Internet and mobile phone delivery vehicles for global diffusion. Curr. Opin. Psychiatr..

[148] Danquah L.O., Hasham N., MacFarlane M., Conteh F.E., Momoh F., Tedesco A.A., Jambai A., Ross D.A., Weiss H.A. (2019). Use of a mobile application for Ebola contact tracing and monitoring in northern Sierra Leone: A proof-of-concept study. BMC Infect. Dis..

[149] Iribarren S.J., Schnall R., Stone P.W., Carballo-Diéguez A. (2016). Smartphone Applications to Support Tuberculosis Prevention and Treatment: Review and Evaluation. JMIR mHealth uHealth.

[150] Menni C., Valdes A.M., Freidin M.B., Sudre C.H., Nguyen L.H., Drew D.A., Ganesh S., Varsavsky T., Cardoso M.J., El-Sayed Moustafa J.S. (2020). Real-time tracking of self-reported symptoms to predict potential COVID-19. Nat. Med..

[151] Drew D.A., Nguyen L.H., Steves C.J., Menni C., Freydin M., Varsavsky T., Sudre C.H., Cardoso M.J., Ourselin S., Wolf J. (2020). Rapid implementation of mobile technology for real-time epidemiology of COVID-19. Science.

[152] Alimohamadi Y., Taghdir M., Sepandi M. (2020). Estimate of the Basic Reproduction Number for COVID-19: A Systematic Review and Meta-analysis. J. Prev. Med. Public Health Yebang Uihakhoe Chi.

[153] Liu Y., Gayle A.A., Wilder-Smith A., Rocklöv J. (2020). The reproductive number of COVID-19 is higher compared to SARS coronavirus. J. Travel Med..

[154] Kobayashi T., Jung S.M., Linton N.M., Kinoshita R., Hayashi K., Miyama T., Anzai A., Yang Y., Yuan B., Akhmetzhanov A.R. (2020). Communicating the Risk of Death from Novel Coronavirus Disease (COVID-19). J. Clin. Med..

[155] Amaro R.E., Mulholland A.J. (2020). A Community Letter Regarding Sharing Biomolecular Simulation Data for COVID-19. J. Chem. Inf. Model..

[156] https://www.trendmicro.com/vinfo/us/security/news/cybercrime-and-digital-threats/coronavirus-used-in-spam-malware-file-names-and-malicious-domains.

[157] Navid Ali K., Sarfraz Nawaz B., Noor Z. (2020). Ten deadly cyber security threats amid COVID-19 pandemic. TechRxiv.

[158] Simko L., Calo R., Roesner F., Kohno T. (2020). COVID-19 Contact Tracing and Privacy: Studying Opinion and Preferences. arXiv.

[159] Cho H., Ippolito D., Yu Y.W. (2020). Contact tracing mobile apps for COVID-19: Privacy considerations and related trade-offs. arXiv.

[160] De Carli A., Franco M., Gassmann A., Killer C., Rodrigues B., Scheid E., Schoenbaechler D., Stiller B. (2020). WeTrace--A Privacy-preserving Mobile COVID-19 Tracing Approach and Application. arXiv.

[161] Ferrara E. (2020). #COVID-19 on Twitter: Bots, Conspiracies, and Social Media Activism. arXiv.

[162] Sen-Crowe B., McKenney M., Elkbuli A. (2020). Social distancing during the COVID-19 pandemic: Staying home save lives. Am. J. Emerg. Med..

[163] Paital B., Das K., Parida S.K. (2020). Inter nation social lockdown versus medical care against COVID-19, a mild environmental insight with special reference to India. Sci. Total Environ..

[164] Rajkumar R.P. (2020). COVID-19 and mental health: A review of the existing literature. Asian J. Psychiatr..

[165] Azoulay P., Jones B. (2020). Beat COVID-19 through innovation. Science.

[166] Chang D., Xu H., Rebaza A., Sharma L., Dela Cruz C.S. (2020). Protecting health-care workers from subclinical coronavirus infection. Lancet. Respir. Med..

[167] Zuniga J.M., Cortes A. (2020). The role of additive manufacturing and antimicrobial polymers in the COVID-19 pandemic. Expert Rev. Med Devices.

[168] Aydemir D., Ulusu N.N. (2020). Correspondence: Angiotensin-converting enzyme 2 coated nanoparticles containing respiratory masks, chewing gums and nasal filters may be used for protection against COVID-19 infection. Travel Med. Infect. Dis..

[169] Smith A.C., Thomas E., Snoswell C.L., Haydon H., Mehrotra A., Clemensen J., Caffery L.J. (2020). Telehealth for global emergencies: Implications for coronavirus disease 2019 (COVID-19). J. Telemed. Telecare.

[170] Bashshur R., Doarn C.R., Frenk J.M., Kvedar J.C., Woolliscroft J.O. (2020). Telemedicine and the COVID-19 Pandemic, Lessons for the Future. Telemed. J. E-Health Off. J. Am. Telemed. Assoc..

[171] Portnoy J., Waller M., Elliott T. (2020). Telemedicine in the Era of COVID-19. J. Allergy Clin. Immunol. Pr..

[172] Neri E., Coppola F., Miele V., Bibbolino C., Grassi R. (2020). Artificial intelligence: Who is responsible for the diagnosis?. La Radiol. Med..

[173] Dean N.R., Rosenthal E.L., Carroll W.R., Kostrzewa J.P., Jones V.L., Desmond R.A., Clemons L., Magnuson J.S. (2010). Robotic-assisted surgery for primary or recurrent oropharyngeal carcinoma. Arch. Otolaryngol. Head Neck Surg..

[174] Kovach C.R., Taneli Y., Neiman T., Dyer E.M., Arzaga A.J.A., Kelber S.T. (2017). Evaluation of an ultraviolet room disinfection protocol to decrease nursing home microbial burden, infection and hospitalization rates. BMC Infect. Dis..

[175] Centers for Disease Control and Prevention, I.G.F.C. Handling, and Testing Clinical Specimens from Persons Under Investigation (PUIs) for Coronavirus Disease 2019 (COVID-19), 9 March 2020. www.cdc.gov/coronavirus/2019-ncov/lab/guidelinesclinical-specimens.html.

